# Powerful and robust non-parametric association testing for microbiome data via a zero-inflated quantile approach (ZINQ)

**DOI:** 10.1186/s40168-021-01129-3

**Published:** 2021-09-02

**Authors:** Wodan Ling, Ni Zhao, Anna M. Plantinga, Lenore J. Launer, Anthony A. Fodor, Katie A. Meyer, Michael C. Wu

**Affiliations:** 1 Public Health Sciences Division, Fred Hutchinson Cancer Research Center, 1100 Fairview Ave N, Seattle, 98109 USA; 2 Department of Biostatistics, Johns Hopkins Bloomberg School of Public Health, 615 N. Wolfe St, Baltimore, 21205 USA; 3 Department of Mathematics and Statistics, Williams College, 18 Hoxsey St., Williamstown, 01267 USA; 4 Laboratory of Epidemiology and Population Science, NIA, NIH, 7201 Wisconsin Ave, Bethesda, 20814 USA; 5 Department of Bioinformatics and Genomics, University of North Carolina at Charlotte, 9201 University City Blvd, Charlotte, 28223 USA; 6 Nutrition Research Institute and Department of Nutrition, University of North Carolina, 500 Laureate Way, Kannapolis, 28081 USA

**Keywords:** Zero-inflated quantile-based approach, Type I error control, Microbiome differential abundance analysis, Heterogeneity

## Abstract

**Background:**

Identification of bacterial taxa associated with diseases, exposures, and other variables of interest offers a more comprehensive understanding of the role of microbes in many conditions. However, despite considerable research in statistical methods for association testing with microbiome data, approaches that are generally applicable remain elusive. Classical tests often do not accommodate the realities of microbiome data, leading to power loss. Approaches tailored for microbiome data depend highly upon the normalization strategies used to handle differential read depth and other data characteristics, and they often have unacceptably high false positive rates, generally due to unsatisfied distributional assumptions. On the other hand, many non-parametric tests suffer from loss of power and may also present difficulties in adjusting for potential covariates. Most extant approaches also fail in the presence of heterogeneous effects. The field needs new non-parametric approaches that are tailored to microbiome data, robust to distributional assumptions, and powerful under heterogeneous effects, while permitting adjustment for covariates.

**Methods:**

As an alternative to existing approaches, we propose a zero-inflated quantile approach (ZINQ), which uses a two-part quantile regression model to accommodate the zero inflation in microbiome data. For a given taxon, ZINQ consists of a valid test in logistic regression to model the zero counts, followed by a series of quantile rank-score based tests on multiple quantiles of the non-zero part with adjustment for the zero inflation. As a regression and quantile-based approach, the method is non-parametric and robust to irregular distributions, while providing an allowance for covariate adjustment. Since no distributional assumptions are made, ZINQ can be applied to data that has been processed under any normalization strategy.

**Results:**

Thorough simulations based on real data across a range of scenarios and application to real data sets show that ZINQ often has equivalent or higher power compared to existing tests even as it offers better control of false positives.

**Conclusions:**

We present ZINQ, a quantile-based association test between microbiota and dichotomous or quantitative clinical variables, providing a powerful and robust alternative for the current microbiome differential abundance analysis.

Video Abstract

**Supplementary Information:**

The online version contains supplementary material available at (10.1186/s40168-021-01129-3).

## Background

High-throughput sequencing technology has enabled large-scale microbiome profiling via 16S rRNA gene amplicon sequencing and shotgun metagenomic sequencing [1]. A recurring objective of human microbiome profiling studies is to identify individual bacterial taxa that are associated with experimental conditions, exposures, or other outcome variables of interest. Such trait-associated taxa (referred to as differentially abundant taxa, for simplicity) can provide clues to the biological mechanisms underlying conditions or responses and facilitate follow-up investigations of the impact of microorganisms on human diseases, leading to novel preventive or therapeutic strategies. Consequently, differential abundance analysis has become a critical step in microbiome studies and has resulted in identification of bacterial taxa related to a wide range of conditions including obesity [2], type 2 diabetes [3], and bacterial vaginosis [4], among others.

Despite many successes, the most appropriate approach to differential abundance analysis is still unclear. Most differential abundance analysis approaches tailored towards genomic and microbiome data assume a statistical distribution for the transformed read counts, causing inflated false positive findings when these assumptions fail. On the other hand, classical statistical methods such as Wilcoxon tests are conservative, controlling type I error but losing power since they fail to fully exploit the data characteristics.

Many papers demonstrate poor type I error control of existing methods [5–7]. Due to the complex distributional attributes of microbiome data (even after normalization), such as sparsity, heavy tails [8], multimodality [9], and other heterogeneity, strong parametric assumptions rarely hold. For example, DESeq2 [10] and edgeR [11, 12] model the read counts using a negative binomial distribution with an offset to account for sequencing depth. Limma-voom [13] models the log counts by a linear model. These approaches can suffer from serious type I error inflation when the (log-transformed) data are far from the negative binomial or linear model. Corncob [14] assumes that the read counts are generated from a beta-binomial distribution, which accommodates some over-dispersion, but may not fully capture the distributions. Moreover, a separate feature of corncob is the ability to test associations between a taxon variability and variables of interest; though, the algorithm often fails to converge in the presence of covariates.

Recognizing the sparsity of the data, many groups have proposed zero-inflated models, which assume the data is distributed as a mixture of zero and a positive distribution (e.g., negative binomial, log-normal, beta, and gamma distributions) [15–19], to specifically account for the biases due to the undersampling of the microbial community [20–22]. For example, metagenomeSeq [18] first normalizes the read counts through cumulative sum scaling (CSS, dividing counts by the total counts up to a fixed quantile in each sample), and subsequently models the data via a zero-inflated Gaussian/log-normal model. Monocle [23, 24] utilizes generalized additive models assuming negative binomial for the positive component, or uses a tobit model (a censored Gaussian linear model) depending on the nature of the normalized counts. Although these methods can potentially offer increased power, they still depend on strong parametric assumptions for the non-zero component of the normalized data, which leads to inflated type I error if the assumptions are not satisfied.

Alternatively, we may first transform the microbiome data, then apply classical statistical methods. This generally helps to control the type I error, but suffers from a loss of power. In this approach, normalization can be done by, for example, dividing the counts by the total sequencing depth (i.e., the total read counts across all taxa in a sample, also referred to as library size) to obtain proportions, or conducting log-ratio (CLR) transformation of the data to mitigate compositionality [25, 26]. Subsequent analyses use classical methods such as linear regression, t-tests, or Wilcoxon tests (which has been repackaged as the LeFSe approach [27]). However, these approaches often struggle with zeroes and ties; many of them cannot adjust for covariates, including Wilcoxon and Kolmogorov–Smirnov (KS) tests; and they lose power by not taking full advantage of data characteristics. Recently, LDM [28] uses the sum of squares decomposition in multivariate linear models to test hypotheses in the microbiome. Though improved from classical methods, it is still underpowered because of the conservative linear model.

In addition, the abundance of normalization methods makes the advantages of existing strategies controversial. The sequencing depth can vary substantially between samples, reflecting only differential efficiency in the sequencing process, not real biological variations. Therefore, it is necessary to normalize the data so that different samples are comparable. Unfortunately, there are various methods, such as rarefying (resampling as if each sample has the same total counts), CSS, total sum scaling (TSS, dividing counts by the sequencing depth), and others, and the performance of some strongly parametric approaches, mainly the tailored approaches for genomic and microbiome data, depends heavily on the normalization choices. For example, DESeq2 internally implements relative log expression (RLE) normalization, and metagenomeSeq requires CSS normalization.

To address the aforementioned challenges, in this paper, we propose a zero-inflated quantile test (ZINQ) for associations between microbiome taxa and a clinical variable (dichotomous or quantitative), achieving robust and powerful inference regardless of the data’s distributional attributes and normalization method. Quantile regression [29] is a robust regression tool that avoids any parametric assumptions. By aggregating the results of quantile regression on multiple quantile levels, e.g., the 1st quartile (*τ*=0.25) and median (*τ*=0.5) of the normalized read counts, we can boost the power by detecting higher-order associations in addition to the mean effect. This will help identify biological mechanisms that affect more than the mean of abundance, such as the dispersion or upper tail of abundance, enabling a comprehensive understanding of heterogeneous microbiome effects.

However, a direct application of quantile regression is problematic due to the sparsity of microbiome data. Quantile regression requires the outcome variable to be purely continuous, which is violated by the presence of many zeroes in microbiome data. Also, it implicitly assumes a constant probability of observing non-zero abundance, failing to account for undersampling biases. As a remedy, ZINQ is derived from a two-part quantile regression model for zero-inflated microbial abundance. It comprises a valid test using logistic regression for the zero counts, and a sequence of novel quantile rank-score based tests for the non-zero part. We make the final decision by combining the marginal *p*-values using a MinP or Cauchy procedure. We demonstrate the performance of ZINQ using real and simulated data, and compare it to the existing differential abundance analysis approaches.

This work provides a robust and powerful non-parametric regression approach to association testing for microbiome data. The first contribution is to incorporate the quantile regression framework into microbiome analysis, relieving the inflated type I error in existing parametric approaches while maintaining the merits of regression, such as adjusting for covariates. Secondly, the test improves power by combining the effect of the investigated variable on both the probability of the taxon being observed and the distribution of its abundance when detected, regardless of the magnitude or direction of the effect. ZINQ’s performance is superior to competitors when the variable’s effect is heterogeneous, for example, diminishing at lower or higher percentiles of the abundance. Finally, it is broadly applicable regardless of the normalization methods. As a non-parametric method, it can handle data after any transformation or without normalization. Therefore, ZINQ enables powerful differential abundance analysis to identify complex biological mechanisms on microbial read counts, while easing the worry about inflated false positives.

## Methods

The fundamental idea underlying our approach is to model the zero inflation and then separately, but non-parametrically, model multiple selected percentiles of non-zero values of the taxon abundance. In this section, we first describe our notation, followed by the proposed two-part quantile model for simultaneous modeling of zeroes and non-zeroes, as well as the formal testing procedure.

### Notation

Suppose the data consist of *n* samples, and from each sample, the counts of *J* taxa are summarized. We then have an *n*×*J* taxon table ***Y***^0^, and the entry $Y^{0}_{i, j}$ denotes the count of the *i*th sample on the *j*th taxon. We denote ***Y*** as the normalized read count table following any normalization method. In this paper, we treat the microbiome data after normalization as the outcome in regression models, and relate them to the clinical variable of interest and other covariates. Note that *Y*_*i*,*j*_ is zero-inflated, and the non-zero part can be either count or continuous depending on the normalization method. Next, each sample has a set of characteristics $\boldsymbol {X}_{i} = (\boldsymbol {Z}_{i}^{\top }, C_{i})^{\top }$, where *C*_*i*_ is the clinical variable under investigation and ***Z***_*i*_ denotes a *p*-vector of other covariates, including the intercept. The objective is to identify which of the *Y*_*j*_’s are associated with *C*, i.e., which taxa are differentially abundant according to *C*. To do this, we will perform a taxon-level analysis for each taxon *j*,*j*=1,…,*J*. Thus, we omit the subscript *j* for a simpler presentation in the rest of this paper.

### Two-part quantile regression model

As a common approach to address zero-inflated outcomes, a two-part [30] or a hurdle model [31] models the chance of observing a positive outcome and the mean of the non-zero outcome separately. We use a similar strategy. First, we assume that the probability of observing a non-zero *Y*_*i*_,*P*(*Y*_*i*_>0|***X***_*i*_), follows a logistic model,

$$\text{logit} \{P (Y_{i} > 0 |\boldsymbol{X}_{i})\} = \boldsymbol{Z}_{i}^{\top} \boldsymbol{\zeta} + \gamma \, C_{i}, $$

where ***ζ*** and *γ* are the true logistic coefficients associated with the covariates and condition of interest. Next, instead of modeling the mean by traditional parametric models, we use linear quantile regression to model the non-zero part, *Y*_*i*_|*Y*_*i*_>0. We assume

$$Q_{Y_{i}} (\tau | \boldsymbol{X}_{i}, Y_{i} > 0) = \boldsymbol{Z}_{i}^{\top} \boldsymbol{\alpha} (\tau) + \beta(\tau) \, C_{i}, $$

where ***α***(*τ*) and *β*(*τ*) are the true quantile coefficients at the *τ*th quantile of non-zero *Y*_*i*_, e.g., $Q_{Y_{i}}(0.5 | \boldsymbol {X}_{i}, Y_{i} > 0)$ is the conditional median and $Q_{Y_{i}}(0.75 | \boldsymbol {X}_{i}, Y_{i} > 0)$ is the conditional 3rd quartile of the non-zero abundance. Note that if *Y* is a count variable, to break the ties and achieve valid inference, we add a perturbation to the outcome, i.e., *W*_*i*_=*Y*_*i*_+*U*, *U*∼*U*(0,1), and model the conditional quantiles of *W*_*i*_ (the standard technique to apply quantile regression for counts [32]). The quantile coefficients ***α***(*τ*) and *β*(*τ*) can be estimated by minimizing the following loss function

1 $$  \min_{\boldsymbol{\alpha},\beta} \sum_{i = 1}^{n} \rho_{\tau}\{Y_{i} - \boldsymbol{Z}_{i}^{\top} {\boldsymbol{\alpha}}- \beta \, C_{i} \} I (Y_{i} > 0),  $$

where *ρ*_*τ*_(*u*)=*u*{*τ*−*I*(*u*<0)} is the standard quantile loss function [33].

In the two-part model, *γ* and *β*(*τ*), *τ*∈(0,1) are jointly of interest, characterizing the association between the variable of interest and the entire distribution of the taxon’s normalized abundance. Specifically, *γ* describes the effect of the variable on the presence and absence of the taxon, and *β*(*τ*) reflects the association of the variable with the distribution of the normalized abundance when the taxon is present in the sample. Thus, our global null hypothesis in the differential analysis is

2 $$  H_{0}: \gamma = 0 \quad \& \quad \beta(\tau) = 0 \,\, \forall \tau \in (0, 1),  $$

such that there is no difference in zero inflation (*γ*=0) nor at the quantiles (*β*(*τ*)=0,*τ*∈(0,1)). Conversely, the alternative hypothesis is *H*_*A*_:*γ*≠0 or *β*(*τ*)≠0 at some percentiles of its abundance indicating that the abundance of the taxon is associated with the variable of interest *C*.

### Zero-inflated quantile rank-score based test (ZINQ)

**Fig. 1:**
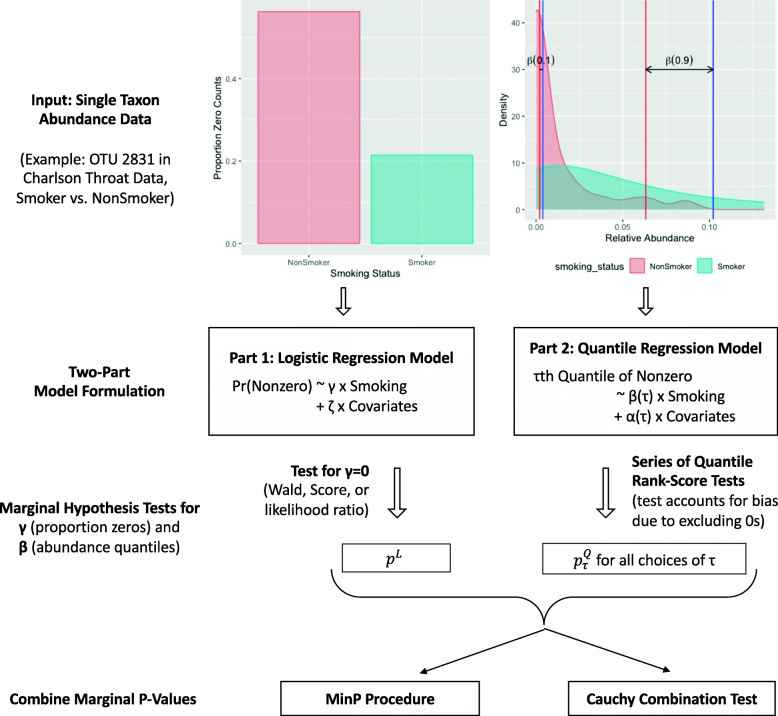
Graphical illustration of the step-wise implementation of ZINQ. Step 1: Test of *γ*=0 by any valid test of logistic regression tells whether the variable of interest is associated with the presence-absence status of the taxon in samples. Step 2: Test of *β*(*τ*_*k*_)=0 by the novel quantile rank-score test adjusting for zero inflation tells whether the variable of interest is associated with the difference at the *τ*_*k*_th percentile of the taxon’s non-zero measurements. The testing is conducted marginally on *K* selected quantiles of the non-zero abundance, such as the default grid. Step 3: Combine the marginal *p*-values in Steps 1 and 2 considering the dependence structure of the tests. Only when the aggregate *p*-value is significant, we conclude that the taxon is differentially abundant

As illustrated in Fig. 1, to test the hypothesis (2), our strategy is first to test *γ*=0, confirming whether there is a difference between the groups concerning the likelihood of the taxon being present in the samples. Independently, we test *β*(*τ*)=0 on the non-zero measurements (accounts for the bias due to excluding zeroes) to see whether the *τ*th percentile of the taxon abundance is different between the groups given it is present. *K* percentiles are chosen to investigate typical locations of the non-zero distribution. Finally, we combine all the marginal *p*-values accounting for the relationships of the tests to avoid type I error inflation by multiple-testing. Only when the summarized *p*-value is significant, we conclude that the taxon is differentially abundant. The detailed algorithm is as follows:

**Step 1**: Conduct any valid test of logistic regression regarding *γ*=0, i.e., Wald test, Rao’s score test or likelihood-ratio test, on the data {(***X***_*i*_,*I*(*Y*_*i*_>0));*i*=1,…,*n*}. Denote the test statistic as *T*^*L*^ and the *p*-value as *p*^*L*^.

**Step 2**: Conduct a sequence of quantile rank-score tests on the subset of non-zero *Y*_*i*_’s regarding *β*(*τ*_*k*_)=0,*k*=1,⋯,*K* (Appendix 1). Denote the test statistics as $T^{Q}_{\tau _{k}}$ and the *p*-values as $p^{Q}_{\tau _{k}}, k = 1, \cdots, K$.

**Step 3**: Combine the marginal *p*-values by the MinP procedure [34, 35] or Cauchy combination test [36].

For the MinP procedure, the smallest *p*-value, $T_{\text {ZINQ-MinP}} = \min \{p^{L}, p^{Q}_{\tau _{1}}, \cdots, p^{Q}_{\tau _{K}}\},$ is the test statistic: we reject the null hypothesis (2) if it is unlikely to observe an even smaller minimum *p*-value. The final *p*-value can be obtained by a resampling method based on the relationships among *T*^*L*^ and $T^{Q}_{\tau _{k}}, k=1, \cdots, K$ (Appendix 2). For the Cauchy combination test, we use $T_{\text {ZINQ-Cauchy}} = \widehat r_{n} \tan \{(0.5 - p^{L}) \pi \} + \sum _{k = 1}^{K} w_{k} \tan \{(0.5 - p^{Q}_{\tau _{k}}) \pi \}$, a weighted sum of the tangent-transformed individual *p*-values as a test statistic. Here, $\widehat r_{n}$ is the observed proportion of zero in *Y*_*i*_’s, and $w_{k} = (1-\widehat r_{n}) \frac { \tau _{k} I (\tau _{k} \leq 0.5) + (1-\tau _{k}) I (\tau _{k} > 0.5) }{\sum _{k=1}^{K} \{\tau _{k} I (\tau _{k} \leq 0.5) + (1-\tau _{k}) I (\tau _{k} > 0.5)\}}$, i.e., the sum of all weights is 1, and the *p*-values for central quantiles are assigned with larger weights while the *p*-values on extreme tails have smaller weights. The final *p*-value can be computed easily as *T*_ZINQ-Cauchy_ converges to the standard Cauchy distribution under the null hypothesis.

Through Steps 1–3, we aggregate the clinical variable’s effect over the distribution of taxon abundance, including the zero counts and various quantiles of the non-zero part. We emphasize that ZINQ (as with LeFSe and the Wilcoxon test) is a global test, in which we are assessing any differences. But in contrast to other global tests, ZINQ has the advantage that we can further evaluate where differences may be observed, providing further clues as to the manner of the association, i.e., whether the overall distribution is shifted or there is some quantile at which there is a substantial difference (indicating a subgroup effect).

### Fine tuning on the grid of quantile levels

The selection of a quantile grid affects the testing performance, so we recommend a fine tuning process. There are two rules for searching. First, to thoroughly investigate the distribution of microbial abundance, we prefer a grid that covers typical locations of the distribution, e.g., the quartiles. Second, to avoid high dependence among marginal results that likely leads to uncontrolled false positives, the number of quantile levels needs to be less than the number of non-zero measurements of the investigated taxon.

Microbiome data is highly sparse and over-dispersed. Thus, there may be a limited number of effective observations for quantile estimation. To be cautious about the type I error, we recommend the conservative default, *τ*=0.1,0.25,0.5,0.75,0.9 (common practice in quantile analysis) for common taxa, and *τ*=0.25,0.5,0.75 for rare taxa. Also, for discrete normalized abundance (e.g., after rarefaction), *τ*=0.25,0.5,0.75 is preferred as the data is even more sparse and extra noise is introduced by perturbation during analysis. Next, as estimates at multiple quantiles are closely related, adding extra quantiles might introduce more signals but also import more noise. Consequently, we recommend that researchers start with the default. If the sample size and taxon abundance permit, they may try a series of finer grids. The final grid choice will be the one that permits detection of differentially abundant taxa at meaningful quantiles while keeping type I error well-controlled.

### Overview of CARDIA data

The Coronary Artery Risk Development in Young Adults (CARDIA [37]) Study enrolled 5115 young adults (ages 18–30) in 1985–1986 with the aim of elucidating risk factors for cardiovascular disease. Subject enrollment was balanced according to Black or white race, gender, education (more than high school or not), and age. Each subject participated in up to eight follow-up visits during 1987–1988 (year 2), 1990–1991 (year), 1992–1993 (year 7), 1995–1996 (year 10), 2000–2001 (year 15), 2005–2006 (year 20), 2010–2011 (year 25), and 2015–2016 (year 30). A variety of factors that are related to cardiovascular disease have been collected, including blood pressure. Physical measurements such as weight and body composition and lifestyle factors such as dietary and exercise patterns have also been collected.

At the Year 30 follow-up examination (2015–2016), stool samples were collected, and the 16S rRNA marker gene was sequenced to obtain the gut microbiota profiles. Sun et al. [38] examined the cross-sectional associations between gut microbial diversity/taxonomic composition and blood pressure. They conducted genus-specific analyses using multiple linear regression with *p*-values adjusted by the Benjamini-Hochberg (BH) procedure. As described by Sun et al., “many individual genera lost statistical significance after adjustment for demographic, health behavior and clinical covariates”. We will use ZINQ to improve their results.

To be consistent with the original study [38], we focused on microbiome count data aggregated at the genus-level and removed genera that were present in less than 25% of participants to avoid spurious findings due to rare genera. The processed data has data on 149 genera for 531 subjects. We aim to find all genera that have cross-sectional associations with the status of having high blood pressure (HBP). The same data from CARDIA will also serve as a basis for our simulation studies. Note that we could quantify blood pressure either as a continuous variable or as a binary variable (HBP vs. non-HBP). For demonstration purposes, we use HBP as a binary variable in this article. Table 1 shows that three covariates [38], age, physical activity score and dietary quality score, are not balanced between participants with and without HBP, suggesting the need to adjust for these variables in the analysis.

**Table 1:** Summary statistics of three important covariates of CARDIA in groups with / without HBP

	Without HBP	With HBP	*p*-value
*N*	346	185	
Age	55.12 (3.44)	55.78 (3.39)	0.034
Physical activity score	393.76 (307.74)	263.84 (241.39)	< 0.001
Dietary quality score	3.43 (5.84)	1.29 (5.39)	< 0.001

*p*-values are calculated by 2-sample *t* test.

Stratified count, mean and standard deviation of age, physical activity score and dietary quality score in groups with/without HBP

### Simulation scenarios

We carried out four simulation studies to assess the type I error control and power of ZINQ in comparison with existing approaches. All simulations used the CARDIA data as the starting data and aimed to identify differentially abundant taxa between subjects with HBP and without HBP.

The first simulation study, named “unadjusted analysis on a single taxon”, investigated the association between four typical taxa (two common and two rare) and HBP without adjustment for other covariates.

In the second simulation, we generated an entire OTU table via the proposed two-part quantile regression model, and assessed the association of each taxon with HBP adjusting for the three covariates in Table 1. We name this simulation “adjusted analysis on an OTU table”.

In the third simulation, to favor mean-based approaches, we generated OTU tables through the Dirichlet-Multinomial (DM) distribution instead of the two-part quantile regression model, and examined the association between each taxon and HBP without adjusting for covariates. We name this simulation scenario “unadjusted analysis on a DM OTU table”.

We also permuted the CARDIA data to create null distributions, and assessed the type I error of association testing between each taxon and HBP adjusting for the covariates in Table 1. The results will be used to assist subsequent real analyses on CARDIA data.

For ZINQ, we considered both MinP and Cauchy procedures for *p*-value combination and used the default quantile grids depending on the specific scenarios.

#### Simulation 1 - unadjusted analysis on a single taxon

For common taxa, we selected two representative genera, *Anaerovorax* and *Saccharibacteria*. *Anaerovorax* is differentially abundant in the processed CARDIA data [38], with strong differences in mean abundance by HBP status. *Saccharibacteria* is not differentially abundant by ordinary linear regression. However, HBP has strong effects on the 1st quartile to the median of the microbe’s abundance (by direct application of quantile regression, perturbing zeroes to break ties). The two genera are examples with a mean association and substantial quantile associations with HBP, respectively.

We simulated the normalized abundance of the two genera from the empirical distribution functions (edf) of their measurements in the normalized CARDIA data by (1) rarefaction, (2) TSS or (3) CSS. We set the sample size as 600 (comparable to CARDIA data), divided evenly between samples with and without HBP (different from the real data, but suitable for type I error and power investigation). To assess type I error control, we simulated null data by generating the 600 samples exclusively from the edf of the normalized abundance in subjects without HBP. To assess power, we created three settings. In setting 1, we generated 300 samples each from the edf of HBP and non-HBP groups so that the effect size is the same as in the real data. In setting 2, for the 300 “with HBP” samples, we generated 80% of them from the HBP edf, while generated the remaining 20% from the non-HBP edf; similarly, we simulated a mixture of 20% HBP measurements and 80% non-HBP ones for “without HBP” samples. In setting 3, we generated mixtures as in setting 2, but changed the proportions to 60% and 40%. As a result, we generated multiple effect sizes by decreasing the true signal strength from setting 1 to 3. We also used sample sizes 50, 100, and 200, with half HBP and half non-HBP samples, to mimic scenarios with more limited sample sizes.

To study the performance of ZINQ on rare taxa, we picked two genera, *Propionispira* and *Corynebacterium*, with prevalence 5% in the CARDIA data before filtering (not included in the processed data). Similarly, *Propionispira* and *Corynebacterium* have mean and quantile associations with HBP, respectively. We simulated their null and alternative data following the same procedures, but only with a sample size of 600 since small sample sizes are likely to result in uniformly zero counts.

We applied 7 parametric zero-inflated methods to the simulated data, in comparison with ZINQ: (1) zero-inflated Poisson (ZIP) for rarefied data, (2) zero-inflated negative binomial (ZINB) for rarefied data, (3) zero-inflated beta regression (ZIB) for TSS normalized data, a popular approach for compositional microbiome data, (4) tobit model (assumes left-censoring at 0) for TSS and CSS normalized data, which is the model of Monocle when the positive normalized data is continuous, (5) zero-inflated log-normal model (ZIlogN) for TSS and CSS normalized data, which is the model of metagenomeSeq, (6) zero-inflated gamma (ZIG) for TSS and CSS normalized data, and (7) linear regression for all three normalization methods. We aimed to use those competing approaches to illustrate the limitations of strong parametric assumptions on microbiome data.

A taxon was considered differentially abundant if the corresponding *p*-value was less than 0.05 or 0.01. The simulation process was repeated 10,000 times. Then, we assessed type I error control on the null data by the percentage of differentially abundant cases over the 10,000 runs, and computed power on the alternative data by the proportion of positive calls among the 10,000 replicates.

#### Simulation 2 - adjusted analysis on an OTU table

We rarefied the CARDIA data 10 times to read depth 46,663 (the minimum read depth in the processed CARDIA data), and averaged the resulting datasets to create the starting data. This multiple rarefaction step was used to avoid highly heterogeneous library sizes among samples and remove bias/randomness in each rarefaction, so as to ensure a proper fitting of models on the starting data. Note that this is not a general normalization procedure and is only used for simulating datasets.

Then, we fitted each of the genera in the starting data by the two-part quantile regression model:

3 $$ \begin{aligned}  \text{logit}\{ P (D=&1 | \boldsymbol{X}) \} = \gamma_{0} + \gamma_{1} \text{HBP} \\ &+ \gamma_{2} \text{age} + \gamma_{3} \text{physical \, activity} \\&+ \gamma_{4} \text{diet \, quality \,score}, \end{aligned}  $$

where *D*=*I*(*Y*>0) is a binary indicator of the presence of the genus and the parameters *γ*_0_,…,*γ*_4_ were estimated from the starting data,

4 $$ {}\begin{aligned}  Q_{Y} (\tau | \boldsymbol{X}, Y > 0) =& \beta_{0}(\tau) + \beta_{1}(\tau)\text{HBP} \\ &+ \beta_{2}(\tau) \text{age} + \beta_{3}(\tau) \text{physical \, activity} \\&+ \beta_{4}(\tau) \text{diet \, quality \,score}, \end{aligned}  $$

where the coefficient functions *β*_0_(*τ*),…,*β*_4_(*τ*),*τ*∈(0,1) were estimated from the non-zero observations of the starting data, using estimates at *τ*=0.01,⋯,0.99 (the fine grid is acceptable to simply estimate quantile functions).

The simulated tables were of the same size as CARDIA data, with 531 samples and 148 genera. To simulate one null OTU table for type I error assessment, we first generated the covariates HBP, age, physical activity and diet quality score for the 531 samples by resampling each of the real covariates with replacement independently (to create “new” samples instead of the real ones in the CARDIA data). Then we generated the read counts based on each of the 148 fitted models for the genera in the CARDIA data, imposing the constraint that *γ*_1_=*β*_1_(*τ*)=0,*τ*∈(0,1). In detail, we simulated the binary indicator *D* by Eq. 3 with *γ*_1_=0. If *D*=0, we assigned 0 as the count. If *D*=1, we simulated the count by the inverse CDF method: randomly drew *U*∼*U*(0,1), computed *Y*=*β*_0_(*U*)+*β*_2_(*U*)physical activity+*β*_3_(*U*)age+*β*_4_(*U*)diet quality score, and rounded it to the nearest integer. To simulate one alternative OTU table for power assessment, we followed the same procedure, but used the fitted models directly without constraints. We also examined OTU tables with 50, 100, and 200 samples, which were generated following the same steps.

We considered four normalization procedures for the simulated OTU tables: (1) no normalization, (2) rarefaction, (3) TSS, or (4) CSS. Then, we applied ZINQ to the four resulting data sets, in comparison with 9 classical and tailored approaches for microbiome analysis: (1) corncob for original and rarefied data, assuming beta-binomial distribution and conducting simultaneous differential abundance and variability analysis, (2) DESeq2 for original and rarefied data, assuming negative binomial distribution, (3) edgeR for original and rarefied data, assuming negative binomial distribution, (4) LDM for all the four data, using linear decomposition model, (5) limma for original and rarefied data, using linear regression on log counts, (6) linear regression for all the four data, (7) metagenomeSeq for CSS normalized data, assuming zero-inflated normal distribution (which is supported by the current algorithm in adjusted analysis, while the log-normal version cannot incorporate covariates besides the variable of interest), (8) Monocle, assuming negative binomial distribution for the original and rarefied data, and tobit model for TSS and CSS normalized data, and (9) QRank [39], a direct quantile approach summarizing a sequence of standard rank-score tests [40] and ignoring zero inflation (perturbation should be added to zeroes to break ties and make algorithm run), for all four data sets. Those competing methods are commonly used in current genomic or microbiome analysis; unlike Wilcoxon or KS tests, they also allow adjustment of covariates, which is suitable for this adjusted analysis. We also considered CLR normalization that removes compositionality of microbiome data, and used applicable methods, LDM, linear regression and QRank to compare with ZINQ. Note that the CLR transformed data can be negative and is continuous without zero inflation, genuinely different from the other normalized data examined in the paper. We also analyzed the CLR transformed data with zeroes filled back.

The taxon was considered differentially abundant if the corresponding *p*-value was less than 0.05 or 0.01. False positive rate (FPR) and true positive rate (TPR) were computed as the proportion of positive calls in one null or alternative OTU table, respectively. As one table contained null or alternative cases exclusively, we regard FPR and TPR as the type I error control and power of the corresponding method. We repeated simulating such null and alternative OTU tables 1000 times, and summarized the average FPR and TPR as the comparison criteria.

#### Simulation 3 - unadjusted analysis on a DM OTU table

To facilitate a fair comparison, we deviated from our proposed model and simulated data based on the DM distribution. This simulation strategy favors the mean-based approaches. We first fitted a DM distribution on the entire starting OTU table (processed in Simulation 2) irrespective of HBP status. We called this model *f*^0^. Next, we fitted two DM models on the stratified starting data consisting of HBP or non-HBP subjects exclusively, called them $f^{1}_{\text {HBP}}$ and $f^{1}_{\text {non-HBP}}$. Note that there are no covariates in the fitted models. Therefore, we did not adjust for them either in the downstream analysis. We repeat all the simulations in Simulation 2.

To simulate one OTU table with 531 (or 50, 100, or 200) samples and 148 genera, we first generated the binary covariate HBP by resampling from the real samples with replacement, and obtained the corresponding library sizes. Then, for a null OTU table, we disregarded the HBP realizations and generated counts of the 148 genera for each sample based on *f*^0^ with the corresponding library size. For an alternative OTU table, we simulated the counts for each sample using $f^{1}_{\text {HBP}}$ when HBP=1, and $f^{1}_{\text {non-HBP}}$ when HBP=0. The same normalization and differential analysis methods in Simulation 2 were used. Average FPR and TPR were summarized over 1000 runs.

#### Simulation 4 – null distribution in permuted CARDIA data

Finally, we assessed type I error control based on permuted CARDIA data and used the results to infer the validity of different approaches in analyzing real CARDIA data. First, we normalized the CARDIA data by (1) rarefaction or (2) CSS. We then permuted the covariates (HBP, age, physical activity score and dietary quality score) jointly over the 531 samples to create a permuted OTU table. Such a permutation maintains the relationships among covariates, but removes the association between HBP and the normalized microbial abundance. Thus, the permuted table should have no differentially abundant taxa, and taxa with small *p*-values are considered false positive signals. We applied ZINQ and all the competing methods in Simulation 2 to the permuted table, then evaluated type I error control by the proportion of taxa with *p*-values less than 0.05. We repeated the process 50 times and summarized the type I errors by boxplots.

## Results

### Type I error and power in Simulation 1

Tables 2 and 3 report the type I error and power in analyzing the genera *Anaerovorax* and *Saccharibacteria* with a sample size of 600, respectively.

**Table 2:** Type I error control and power on simulated data based on *Anaerovorax*’s normalized abundance

Sample size = 600					
	Type I error		Power		
	Null		Setting 1	Setting 2	Setting 3
*α*-level	0.05	0.01	0.05	0.05	0.05
*Rarefaction*					
Linear regression	0.0547	0.0084	0.9949	0.6928	0.1247
ZIP	0.7387	0.6622	1.0000 ^∗^	0.9742 ^∗^	0.7720 ^∗^
ZINB	0.2019	0.0771	0.9812 ^∗^	0.7321 ^∗^	0.2398 ^∗^
ZINQ-MinP	0.0526	0.0106	0.9994	0.8557	0.1508
ZINQ-Cauchy	0.0580	0.0110	0.9991	0.8346	0.1493
*TSS*					
Linear regression	0.0536	0.0088	0.9970	0.7425	0.1320
ZIB	0.0110	0.0017	0.9964 ^+^	0.6255 ^+^	0.0305 ^+^
Tobit	0.0543	0.0099	0.9989	0.8041	0.1467
ZIlogN	0.6992	0.6872	1.0000 ^∗^	1.0000 ^∗^	0.9999 ^∗^
ZIG	0.0548	0.0102	0.9961	0.7264	0.1196
ZINQ-MinP	0.0501	0.0101	0.9995	0.9096	0.1669
ZINQ-Cauchy	0.0503	0.0103	0.9994	0.8981	0.1555
*CSS*					
Linear regression	0.0527	0.0113	0.9995	0.8934	0.1733
Tobit	0.0526	0.0110	0.9985	0.8597	0.1628
ZIlogN	0.0475	0.0095	0.9996	0.8794	0.1464
ZIG	0.0494	0.0096	0.9998	0.8850	0.1474
ZINQ-MinP	0.0501	0.0103	0.9993	0.8852	0.1520
ZINQ-Cauchy	0.0505	0.0095	0.9991	0.8735	0.1524

Setting 1: 100% from HBP edf for HBP samples;

Setting 2: 80% from HBP edf and 20% from non-HBP edf for HBP samples;

Setting 3: 60% from HBP edf and 40% from non-HBP edf for HBP samples.

^∗^: power of a method that inflates type I error

^+^: power of a method that deflates type I error

Results by the various methods on 10000 simulated datasets by generating samples from the edf of *Anaerovorax*’s normalized abundance, including type I error control and power under different settings with significance cutoffs 0.05 and 0.01

**Table 3:** Type I error control and power on simulated data based on *Saccharibacteria*’s normalized abundance

Sample size = 600					
	Type I error		Power		
	Null		Setting 1	Setting 2	Setting 3
*α*-level	0.05	0.01	0.05	0.05	0.05
*Rarefaction*					
Linear regression	0.0247	0.0032	0.0602 ^+^	0.0424 ^+^	0.0326 ^+^
ZIP	0.8238	0.7766	0.8056 ^∗^	0.7642 ^∗^	0.7384 ^∗^
ZINB	0.4241	0.2916	0.3515 ^∗^	0.3304 ^∗^	0.3150 ^∗^
ZINQ-MinP	0.0471	0.0089	0.9243	0.4867	0.0819
ZINQ-Cauchy	0.0506	0.0100	0.9166	0.5428	0.0954
*TSS*					
Linear regression	0.0279	0.0030	0.0372 ^+^	0.0338 ^+^	0.0320 ^+^
ZIB	0.0053	0.0009	0.0649 ^+^	0.0190 ^+^	0.0067 ^+^
Tobit	0.0522	0.0137	0.0837	0.0703	0.0635
ZIlogN	0.9997	0.9997	0.9987 ^∗^	0.9978 ^∗^	0.9983 ^∗^
ZIG	0.0495	0.0073	0.1094	0.0675	0.0498
ZINQ-MinP	0.0428	0.0083	0.6626	0.2480	0.0596
ZINQ-Cauchy	0.0497	0.0099	0.6800	0.2818	0.0700
*CSS*					
Linear regression	0.0500	0.0107	0.2021	0.1034	0.0541
Tobit	0.0498	0.0111	0.1677	0.0929	0.0533
ZIlogN	0.0446	0.0071	0.4933	0.2063	0.0621
ZIG	0.0443	0.0076	0.5563	0.2264	0.0643
ZINQ-MinP	0.0456	0.0085	0.8442	0.3766	0.0720
ZINQ-Cauchy	0.0497	0.0099	0.8327	0.3897	0.0773

Setting 1: 100% from HBP edf for HBP samples;

Setting 2: 80% from HBP edf and 20% from non-HBP edf for HBP samples;

Setting 3: 60% from HBP edf and 40% from non-HBP edf for HBP samples.

^∗^: power of a method that inflates type I error

^+^: power of a method that deflates type I error

Results by the various methods on 10000 simulated datasets by generating samples from the edf of *Saccharibacteria*’s normalized abundance, including type I error control and power under different settings with significance cutoffs 0.05 and 0.01

From Table 2, we see that for a genus having a strong mean association with HBP, ZINQ, using either MinP or Cauchy *p*-value combinations, has well-controlled type I error and demonstrates similar or higher power compared to existing methods, regardless of how the data is normalized. For rarefied data, ZINB has inflated type I errors with 20% of the null taxa having *p*-values less than 0.05 and 8% of them having *p*-values less than 0.01. ZIP performs even worse. In comparison, ZINQ-MinP and ZINQ-Cauchy have type I error rates close to the nominal value of 0.05 and 0.01. ZINQ is even more powerful than ZINB in Setting 2, with more than 83% of the true differentially abundant taxa detected, compared to 73% for ZINB. Linear regression controls type I error well, but inferior to ZINQ in Setting 2 with less than 70% true differentially abundant taxa detected. On the compositional data normalized by TSS, ZIB has deflated type I error with only 1% null taxa having *p*-values less than 0.05, and ZIlogN has inflated type I error with 70% of the null taxa having *p*-values less than 0.05. The remaining approaches, linear regression, Tobit, ZIG and ZINQ, all control type I error well. In terms of power, ZINQ dominates the others in Setting 2, where ZINQ identifies more than 89% of the true differentially abundant taxa, while the first runner-up, Tobit, detects 80% of them. As the CSS normalized data is quite regular, all the approaches have well-controlled type I error, and ZINQ shows similar powers to the competing methods in all the three settings. When sample size is 50 (Additional file 1: Table S1), the type I error of ZINQ is deflated. For sample sizes 100 and 200 (Additional file 1: Tables S2 and S3), ZINQ maintains a proper type I error across different normalizations, and its power is comparable to the existing methods.

From Table 3, we see that for a genus having substantial quantile associations but no mean association with HBP, the merits of ZINQ are amplified. Similar comparison results are seen for rarefied and TSS normalized data, where ZINQ has already shown advantages for *Anaerovorax*, and the improvement is mainly in CSS normalized data. All methods control type I error well on the CSS normalized data, while ZINQ demonstrates superior power to the others. In the three settings, ZINQ has powers more than 83%, 37%, and 7%, respectively, while powers of the first runner-up, ZIG, are only 56%, 23%, and 6%. We again observe type I error deflation of ZINQ when sample size is 50 (Additional file 1: Table S4). When sample size is 100 or 200 (Additional file 1: Tables S5 and S6), ZINQ’s advantages on such taxa having heterogeneous associations with the variable of interest are clearer. It keeps false positives below the nominal levels, and has higher power than the others.

From Table S7 (Additional file 1), we see that for a rare genus with mean differences, ZINQ obtains the nominal significance level, and shows equivalent or higher power than competing approaches. Table S8 (Additional file 1) suggests that the superiority of ZINQ on a genus with quantile differences is robust to the its rarity.

We note that ZINQ-MinP and ZINQ-Cauchy are generally comparable in the single taxon simulation, while MinP procedure is not as stable when sample size is limited, more likely to experience type I error deflation.

Overall, for taxa with either mean or quantile associations with the variable of interest, and when there are reasonably abundant non-zero measurements, ZINQ is robust, controlling type I error well, and shows similar or improved power in detecting differentially abundant taxa regardless of the normalization method.

### Type I error and power in Simulation 2

Table 4 reports the average FPR and TPR of adjusted analysis on 1000 simulated OTU tables generated by the proposed two-part quantile regression model with sample size 531. ZINQ demonstrates a stable control of type I error regardless of how the OTU table was processed and gives the highest power among the valid approaches.

**Table 4:** Average FPR and TPR by adjusted analysis on un-normalized/normalized simulated OTU tables

Sample size = 531								
	*Count*		*Rarefaction*		*TSS*		*CSS*	
*α*-level	0.05	0.01	0.05	0.01	0.05	0.01	0.05	0.01
	FPR							
Corncob	0.1077	0.0498	0.0919	0.0400	-	-	-	-
DESeq2	0.0921	0.0395	0.0779	0.0312	-	-	-	-
EdgeR	0.1034	0.0415	0.0893	0.0331	-	-	-	-
LDM	0.0501	0.0096	0.0499	0.0096	0.0501	0.0096	0.0489	0.0095
Limma	0.0561	0.0128	0.0719	0.0203	-	-	-	-
Linear regression	0.0475	0.0085	0.0469	0.0083	0.0472	0.0083	0.0488	0.0098
MetagenomeSeq	-	-	-	-	-	-	0.1539	0.0759
Monocle	0.7261	0.6695	0.6493	0.5839	0.0486	0.0086	0.0501	0.0102
QRank	0.0493	0.0101	0.0499	0.0100	0.0503	0.0098	0.0496	0.0099
ZINQ-MinP	0.0483	0.0094	0.0484	0.0096	0.0488	0.0096	0.0472	0.0091
ZINQ-Cauchy	0.0533	0.0107	0.0535	0.0106	0.0539	0.0104	0.0530	0.0109
	TPR							
Corncob	0.4544 ^∗^	0.3093 ^∗^	0.4018 ^∗^	0.2615 ^∗^	-	-	-	-
DESeq2	0.3289 ^∗^	0.2346 ^∗^	0.2859 ^∗^	0.1912 ^∗^	-	-	-	-
EdgeR	0.4046 ^∗^	0.2782 ^∗^	0.3653 ^∗^	0.2395 ^∗^	-	-	-	-
LDM	0.3283	0.1850	0.3094	0.1677	0.3283	0.1850	0.4150	0.2700
Limma	0.3981	0.2636 ^∗^	0.3701 ^∗^	0.2369 ^∗^	-	-	-	-
Linear regression	0.3358	0.1867	0.3030	0.1573	0.3214	0.1735	0.4080	0.2735
MetagenomeSeq	-	-	-	-	-	-	0.4900 ^∗^	0.3731 ^∗^
Monocle	0.8637 ^∗^	0.8275 ^∗^	0.8055 ^∗^	0.7579 ^∗^	0.3251	0.1766	0.4107	0.2761
QRank	0.3887	0.2346	0.2981	0.1641	0.3634	0.2160	0.3593	0.2117
ZINQ-MinP	0.4437	0.2733	0.3452	0.1945	0.4188	0.2535	0.4176	0.2519
ZINQ-Cauchy	0.4919	0.3156	0.3941	0.2333	0.4666	0.2943	0.4627	0.2919

^∗^: power of a method that inflates type I error

^+^: power of a method that deflates type I error

Results by the various methods on un-normalized/normalized simulated OTU table generated from the proposed two-part quantile model fitted on CARDIA data, including the average FPR and average TPR over 1000 runs according to significance cutoffs 0.05 and 0.01

Specifically, for the raw read counts, corncob, DESeq2, edgeR and Monocle have inflated type I error, with more than 9% of *p*-values less than 0.05 and more than 4% of *p*-values less than 0.01. Note that when adjusting for covariates, the algorithm of corncob sometimes fails to converge and could produce results for only 2/3 of the taxa simulated. Compared to the remaining valid methods, LDM, limma, linear regression and QRank, all of which have less than 40% power, ZINQ-Cauchy has superior power with more than 49% of the true differentially abundant taxa identified at a significance level of *α*=0.05. ZINQ-MinP is less powerful (44%) but still advantageous.

On the rarefied table, corncob, DESeq2, edgeR, limma, and Monocle fail to control the false positives with more than 7% and 2% of *p*-values less than 0.05 and 0.01, respectively. In terms of power, the valid approaches, LDM, linear regression and QRank, have powers around 30%, while ZINQ-Cauchy and ZINQ-MinP detect more than 39% and 34% of true differentially abundant taxa with the cutoff 0.05.

On compositional data normalized using TSS, all of the candidate methods control type I error well, while ZINQ-Cauchy shows the highest power of 46% (*α*=0.05) and 29% (*α*=0.01) and ZINQ-MinP shows power of 42% and 25%. The first runner-up, QRank, shows power of only 36% and 22%.

For the CSS normalized data, the state-of-the-art approach, metagenomeSeq, cannot control type I error, with 15% and 8% of its *p*-values less than 0.05 and 0.01, respectively. Among the methods that have proper type I error control, ZINQ-Cauchy is the most powerful, finding more than 46% of the true differentially abundant taxa with the cutoff 0.05, ZINQ-MinP shows power of 42%, while the competing approaches have powers less than 42%.

For the CLR normalized data (Additional file 1: Table S9), we see qualitatively similar results as on other normalized data – ZINQ controls type I error and shows power gain. As the major difference of ZINQ from QRank is that it considers zero inflation, we note that QRank is comparable to ZINQ on the CLR normalized data but becomes inferior when zeroes are added back.

When sample size is 50, ZINQ-MinP sometimes inflates its type I error (by TSS, Additional file 1: Table S10). For sample size 100 or 200 (Additional file 1: Tables S11 and S12), both ZINQ-MinP and ZINQ-Cauchy are robust, obtaining nominal significance levels, and demonstrate equivalent power to existing approaches on CSS normalized OTU tables and improved power on other types of data. Comparing the MinP and Cauchy procedures, we see that ZINQ-Cauchy is more robust to small sample size and is marginally more powerful on an OTU table.

To sum up, on an OTU table consisting of hundreds of taxa with various distributional attributes, the non-parametric ZINQ is robust and effectively controls false positives, as long as the samples with non-zero counts are adequate. Among the approaches with proper type I error, ZINQ shows comparable or improved power due to its ability to detect higher-order associations, not just the mean effects.

### Type I error and power in Simulation 3

Table 5 reports the average FPR and TPR of unadjusted analysis on 1000 simulated OTU tables from the DM models with sample size 531. Again, ZINQ controls type I error well no matter how the OTU tables were normalized.

**Table 5:** Average FPR and TPR by unadjusted analysis on un-normalized/normalized simulated DM OTU tables

Sample size = 531								
	*Count*		*Rarefaction*		*TSS*		*CSS*	
*α*-level	0.05	0.01	0.05	0.01	0.05	0.01	0.05	0.01
	FPR							
Corncob	0.0522	0.0115	0.0523	0.0115	-	-	-	-
DESeq2	0.0954	0.0305	0.0951	0.0304	-	-	-	-
EdgeR	0.0588	0.0130	0.0580	0.0133	-	-	-	-
LDM	0.0494	0.0097	0.0493	0.0098	0.0494	0.0097	0.0499	0.0097
Limma	0.0493	0.0098	0.0493	0.0101	-	-	-	-
Linear regression	0.0475	0.0085	0.0475	0.0085	0.0475	0.0085	0.0496	0.0101
MetagenomeSeq	-	-	-	-	-	-	0.1354	0.0552
Monocle	0.9463	0.9296	0.9452	0.9283	0.0481	0.0087	0.0501	0.0102
QRank	0.0489	0.0098	0.0496	0.0096	0.0489	0.0097	0.0491	0.0096
ZINQ-MinP	0.0468	0.0092	0.0468	0.0091	0.0466	0.0090	0.0478	0.0092
ZINQ-Cauchy	0.0522	0.0108	0.0523	0.0106	0.0524	0.0107	0.0523	0.0108
	TPR							
Corncob	0.3009	0.1678	0.3000	0.1675	-	-	-	-
DESeq2	0.2210 ^∗^	0.1095 ^∗^	0.2207 ^∗^	0.1093 ^∗^	-	-	-	-
EdgeR	0.1603	0.0642 ^∗^	0.1610	0.0652 ^∗^	-	-	-	-
LDM	0.1554	0.0646	0.1553	0.0646	0.1554	0.0646	0.2775	0.1530
Limma	0.2923	0.1647	0.2918	0.1646	-	-	-	-
Linear regression	0.1529	0.0611	0.1528	0.0611	0.1528	0.0610	0.2888	0.1619
MetagenomeSeq	-	-	-	-	-	-	0.3165 ^∗^	0.1955 ^∗^
Monocle	0.9610 ^∗^	0.9485 ^∗^	0.9603 ^∗^	0.9476 ^∗^	0.1537	0.0616	0.2901	0.1630
QRank	0.2318	0.1156	0.2316	0.1152	0.2325	0.1162	0.2253	0.1088
ZINQ-MinP	0.2419	0.1244	0.2414	0.1236	0.2422	0.1242	0.2391	0.1194
ZINQ-Cauchy	0.2820	0.1511	0.2814	0.1506	0.2819	0.1514	0.2785	0.1449

^∗^: power of a method that inflates type I error

Results by the various methods on un-normalized/normalized simulated OTU table generated from the DM models fitted on CARDIA data, including the average FPR and average TPR over 1000 runs according to significance cutoffs 0.05 and 0.01

The data was simulated to have definite mean differences between the HBP and non-HBP groups, and the analysis is simple with no covariates. As expected, the mean-based approaches such as limma (on un-normalized and rarefied data), LDM, linear regression and Monocle (on CSS normalized data) show high power. Corncob controls FPR and presents high power on un-normalized and rarefied data, but has inflated type I error when sample size is 50 or 100 (Additional file 1: Tables S14 and S15). Even though the setting was not designed to favor quantile-based methods, ZINQ-Cauchy always demonstrates a top-tier power. Though ZINQ-MinP shows a power reduction compared to those approaches on certain normalized data, none of them shows consistent power gain across all normalization methods. We see similar results when sample size is 50 to 200 (Additional file 1: Tables S14-S16). On the CLR normalized DM OTU table (Additional file 1: Table S13), ZINQ maintains its advantages as in Simulation 2.

Again, on an OTU table that includes taxa with various distributional attributes, though generated with mean associations from DM models, ZINQ-Cauchy is marginally more powerful than ZINQ-MinP.

Therefore, even when the true difference lies in the mean abundance, with adequate non-zero measurements, ZINQ is reliable and robust. It controls type I error and demonstrates a high power regardless of the simulation setup and data preprocessing procedures.

### Type I error in Simulation 4

**Fig. 2:**
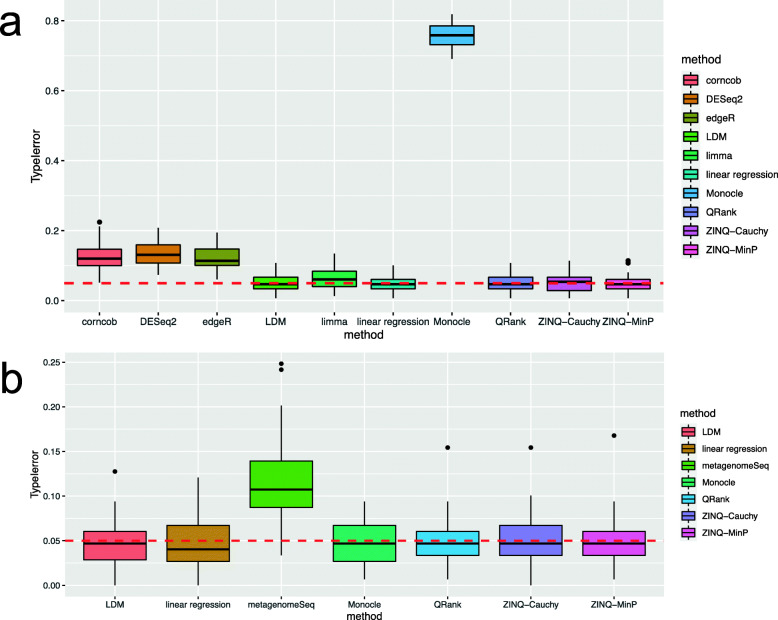
Type I error control of the various methods on permuted normalized CARDIA data. **a** Boxplots of type I error over 50 null rarefied data. **b** Boxplots of type I error over 50 null CSS normalized data

Figure 2 reports the type I errors of various approaches in analyzing permuted normalized CARDIA data. In the top panel, corncob, DESeq2, edgeR and Monocle have inflated type I error with more than 15% *p*-values less than 0.05. Of them, DESeq2, edgeR, and Monocle assume a negative binomial distribution and corncob uses a beta-binomial distribution, suggesting a failure to fully model the microbiome data even with such complex parametric models. The bottom panel of Fig. 2 suggests that after CSS normalization, all the methods have controlled type I error except metagenomeSeq. This investigation provides a list of valid approaches for subsequent analyses of the real CARDIA data.

### CARDIA data analysis

We applied the methods in Simulation 2, and focused on those have proper type I error control in the permuted CARDIA data to study the rarefied and CSS normalized CARDIA data. Taxa were considered differentially abundant if the corresponding BH-adjusted *p*-values were less than 0.05. Table 6 reports the number of differentially abundant taxa detected by the different approaches. It shows that ZINQ is the most powerful, detecting the largest number of differentially abundant taxa among the tests that control type I error, regardless of the normalization method. Note that we picked ZINQ-Cauchy to represent ZINQ, comparing with the others in this section, as it is more powerful than ZINQ-MinP on the CARDIA data (consistent with findings in Simulations 2 and 3).

**Table 6:** Numbers of differentially abundant taxa by valid methods on data normalized by rarefaction or CSS

	Rarefaction	CSS
Corncob	16 ^∗^	–
DESeq2	34 ^∗^	–
EdgeR	33 ^∗^	–
LDM	11	23
Limma	24	–
Linear regression	5	25
MetagenomeSeq	–	40 ^∗^
Monocle	121 ^∗^	20
QRank	13	12
ZINQ-MinP	48	37
ZINQ-Cauchy	**49**	**41**

^∗^: method that inflates type I error

On rarefied data, the valid competing methods, LDM, limma, linear regression and QRank (by Fig. 2) detect 11, 24, 5, and 13 differentially abundant taxa, respectively. In comparison, ZINQ identifies 49 differentially abundant taxa, demonstrating dominating power. On CSS normalized data, ZINQ claims 41 differentially abundant taxa, and the first runner-up among all valid competing approaches (by Fig. 2), linear regression, finds only 25 differentially abundant taxa. Therefore, we can conclude that ZINQ controls false positives well and improves the power in detecting differentially abundant taxa on CARDIA data.

**Fig. 3:**
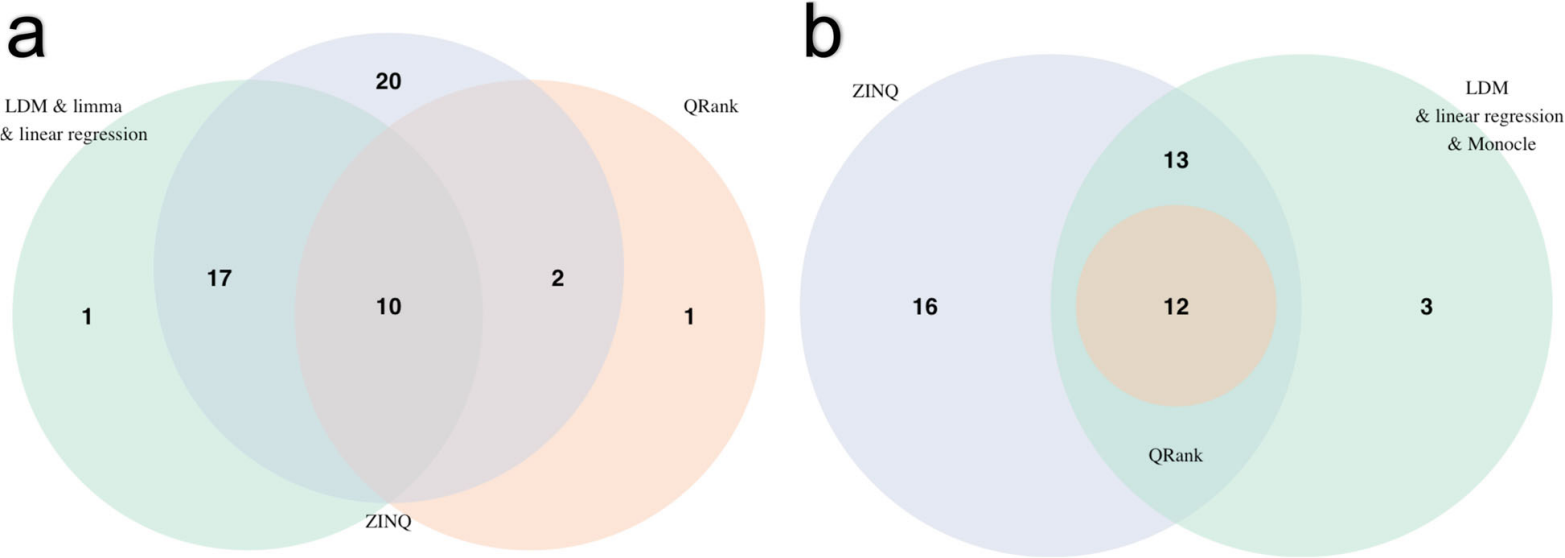
Venn diagrams of the differentially abundant taxa detected by ZINQ, QRank, and valid parametric methods that control type I error. **a** Results on rarefied data and **b** results on CSS normalized data

Figure 3 reports how the numbers of differentially abundant taxa detected by the valid methods overlap with each other. To compare with ZINQ, we grouped the valid parametric methods and considered the results of QRank separately, as these two groups are fundamentally different due to their parametric versus non-parametric nature. On the rarefied data (Fig. 3, left), ZINQ identifies all genera but one found by the valid parametric methods, LDM, limma and linear regression. Also, all of the genera but one detected by QRank are identified by ZINQ. On the other hand, ZINQ exclusively detects 20 genera. On the CSS normalized data (Fig. 3, right), we see similar results: ZINQ detects all except three genera found by LDM, linear regression and Monocle, whereas the two parametric methods fail to identify 16 genera detected by ZINQ. The findings confirm that ZINQ is the most powerful among the approaches that control type I error. It possesses both robustness and high power as it considers zero inflation in a quantile-based approach. As a result, most of the genera detected by the parametric and non-parametric competing methods are also identified by ZINQ, while there is a noticeable number of genera uniquely detected by ZINQ.

**Fig. 4:**
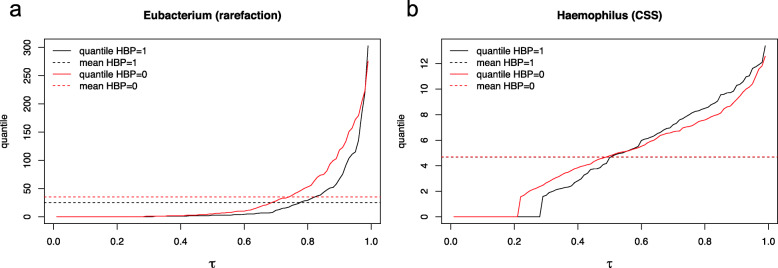
Empirical quantile functions (quantiles of normalized abundance (quantile) vs. quantile levels (*τ*)) stratified by with / without HBP for two typical taxa detected by ZINQ exclusively, with dashed horizontal lines indicating the two group means, which are close or identical in the examples. **a** Spindle shape, HBP is associated with lower *Eubacterium* abundance (rarefied) when the microbe is abundant. **b** Crossing, HBP is associated with lower *Haemophilus* abundance (CSS normalized) when the microbe is at a low level, but with higher abundance when the microbe is abundant

We then investigated the abundance profiles of those genera exclusively identified by ZINQ, and found two patterns that highlight ZINQ’s improved power. We examined two representative genera that correspond to the two patterns in Fig. 4. For both *Eubacterium* (rarefied) and *Haemophilus* (CSS normalized), the mean normalized abundance is nearly the same in the HBP and non-HBP groups, however, the quantiles of their normalized abundance in the two groups are substantially different. The two genera exhibit different patterns of quantile differences.

The quantile functions corresponding to *Eubacterium* in HBP and non-HBP subjects form a spindle shape (Fig. 4, left). The two curves differ between the 60th and 95th percentiles, with the maximum difference attained at the 80th percentile. This finding suggests that when *Eubacterium* is abundant in the gut, having HBP is associated with lower *Eubacterium* abundances.

For *Haemophilus*, the two quantile functions cross each other at the 48th percentile (Fig. 4, right). Thus, in addition to varying in magnitude, the effect of HBP on *Haemophilus* changes direction as well. Biologically, we can conclude that for people with a normal amount of *Haemophilus* in the gut, having HBP or not is unassociated with the abundance of the microbe. However, for subjects with a low level of *Haemophilus*, having HBP is associated with still lower abundances, whereas the opposite is true for subjects with high *Haemophilus*. That is, HBP is associated with more extreme values of *Haemophilus* abundance in both directions, relative to subjects without HBP. This diverse association depending on the abundance level might be driven by the differences in species and strain level effects. Some species dominates at the low abundance level, and associates with HBP in one direction, while another species dominates at the high abundance level and responds in the opposite way. Another example of such a diverse association is *Lactobacillus*[41], which has been observed by most vaginal microbiome researchers. *L. iners*and *L. crispatus*are the most common species and can both dominate the vaginal microbiome, but *L. iners*more often co-occurs with a diverse state associated with bacterial vaginosis. Due to the diversity of effect at the species level, the association with bacterial vaginosis is obscured at the genus level.

From the Venn diagrams and visual investigation of quantile functions, we know that ZINQ can not only detect most of the cases with homogeneous covariate effect/mean difference, but is capable of identifying heterogeneous covariate effect/quantile differences. To validate this claim, we checked the degree of heterogeneity of the microbial abundance-HBP association in the genera exclusively detected by ZINQ or valid parametric methods on CSS normalized CARDIA data (Fig. 3, right). We used the coefficient of variation of the coefficients associated with HBP as the measure of heterogeneity. For each genus, we first computed the logistic coefficient *γ*_1_ in (3) for the zero counts and 19 quantile coefficients *β*_1_(*τ*_*k*_) in (4) with *τ*_*k*_=0.05,⋯,0.95 on the non-zero part. Then, we calculated the absolute value of the ratio between the standard deviation and the mean of those coefficients. Intuitively, a higher value of the coefficient of variation reflects a higher degree of heterogeneity in the microbial abundance-HBP association.

**Fig. 5:**
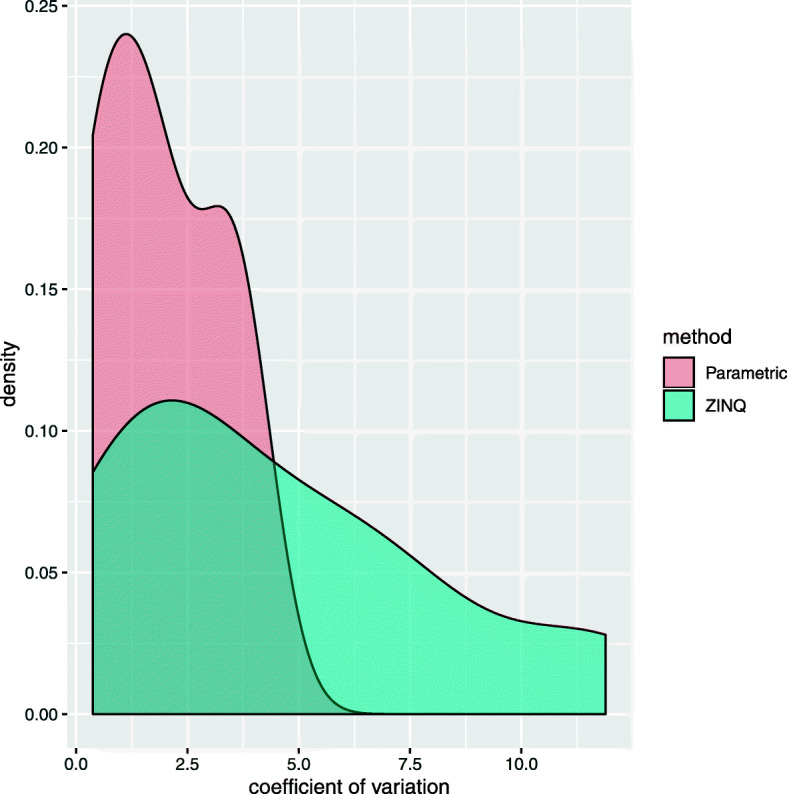
Heterogeneity comparison between the taxa detected by ZINQ exclusively and those found by the valid parametric methods that control type I error but not ZINQ on the CSS normalized data

Figure 5 presents the density plots of the heterogeneity measure in the 16 genera uniquely detected by ZINQ and 3 other genera exclusively identified by the valid parametric methods, linear regression and Monocle. We see that the associations in ZINQ-detected genera are much more heterogeneous than those detected by mean-based parametric approaches. This finding analytically supports our claim that as a non-parametric method, ZINQ cannot be as sensitive as those parametric ones when there is a subtle mean effect of HBP; however, it is more powerful when the signal is heterogeneous, which is prevalent in microbiome data which has complex distributional attributes.

As computation cost is crucial for differential abundance analysis, we summarized the time and memory used by each method to analyze the CARDIA data 10 times. As Table S17 (Additional file 1) suggests, ZINQ is fairly fast and economical. ZINQ-Cauchy entails 4.5 min to analyze the real dataset 10 times, almost the same as the time cost by QRank, which uses standard quantile regression. The recently developed approaches tailored for microbiome data, corncob and LDM, are much slower than ZINQ. In terms of memory, ZINQ-Cauchy uses as much as most of the established differential analysis methods. ZINQ-MinP costs more resources than ZINQ-Cauchy due to its resampling step.

## Discussion

In this paper, we proposed to use a zero-inflated quantile rank-score based test (ZINQ) under a two-part quantile regression model for microbiome differential abundance analysis. The tool detects the difference in zero counts by logistic regression and searches for signals on the non-zero normalized abundance via quantile rank-score based tests. The final testing decision is based on the combined *p*-value of those marginal tests using the MinP procedure or Cauchy combination test. The novel approach controls type I error due to its non-parametric nature that handles various complex distributions robustly. In addition, by examining multiple quantiles of the non-zero abundance, ZINQ improves the testing power by detecting quantile/higher-order associations between the clinical variable and microbial abundance, besides the mean association. Next, as a regression-based method, it is flexible to adjust for covariates. Finally, thanks to its non-parametric nature, ZINQ is generic, applicable to microbiome data processed by any normalization method.

Through simulations and application to the CARDIA data, ZINQ complements and often offers improvements over a number of existing methods, particularly with regard to improved type I error control. Improvements in type I error are, as discussed, often due to the non-restrictive nature of quantile regression towards the underlying distribution. Regarding power, among methods that usually control type I error, ZINQ is most advantageous for taxa for which there are a reasonable number of non-zero counts and may have heterogeneous effects. Under these scenarios, ZINQ uniformly dominates competing approaches. On the other hand, in situations where there are primarily mean effects, competing approaches such as LDM, may have higher power—though ZINQ is usually not too far behind. As large-sample microbiome data become increasingly available thanks to advances in technology, the advantage of ZINQ to detect subtle and heterogeneous differences while adjusting for crucial clinical covariates becomes important. It will help identify complicated biological mechanisms of diseases/exposures on microbes, rather than a simple effect, such as an increase/a decrease of abundance in all people.

In general, ZINQ can be applied to low-frequency taxa as well as more common taxa. However, for low-frequency taxa, we suggest using ZINQ-Cauchy and restricting the quantile levels to central ones, such as *τ*=0.2,0.4,0.5,0.6,0.8, the quartiles or even just the median. Here, “low-frequency” is an operational term that depends upon sample size: taxa with a prevalence of 5% will be observed 50 times if the sample size is 1000, but only 2–3 times if the sample size is 50. In practice, a threshold of 15 non-zero observations may be sufficient to apply ZINQ (given there is no high-dimensional problem due to too many covariates), regardless of sample size. The reason behind this restriction is that if the number of non-zero measurements is small (a concern for small sample sizes), then quantile regression is not stable at quantiles far from the median of the distribution (i.e., the 10th or 90th percentiles). Similarly, ZINQ-Cauchy approach tends to offer better error control for low-frequency taxa due to its finite sample characteristics. Accordingly, for low-frequency taxa below the threshold, specially tailored approaches such as the LDM may offer improvements.

A characteristic of ZINQ is that it can, in principle, be applied to any normalization or transformation of the original count or relative abundance data. Our results demonstrate that it often produces qualitatively similar results across different normalizations: when analyzing the CARDIA data, ZINQ-Cauchy detected 49 and 41 differentially abundant taxa on rarefied and CSS normalized data, respectively, and most of them overlap. The discrepancy only occurs for a taxon when the effect size is small and its statistical significance is borderline.

Despite the many strengths of ZINQ, it does not serve as a panacea for all issues in microbiome association analysis. For example, when library size is heavily confounded with the variable of interest, as with other approaches that consider zero inflation, ZINQ cannot determine whether differences in proportions of zero are due to the variable or caused by the imbalanced sequence depths. For this case, incorporate library size as a covariate in ZINQ may help, though we would suggest using approaches that treat the relative abundance quantitatively, such as linear regression or LDM.

There are various directions to extend ZINQ. First, as the taxa in microbiome data are highly correlated, we can incorporate information from others when analyzing one taxon to achieve a more meaningful and possibly more powerful result. Second, to save computational cost, we can develop an efficient procedure to select the optimal grid of quantile levels.

We only compare approaches under the same normalization in this paper. If one is interested in consolidating results across various normalizations, to control false positive calls, we suggest constructing an omnibus test using the MinP or Cauchy combination approaches. This entails analyzing the data under multiple normalizations methods and then combining the *p*-values for each taxon under the different normalizations.

## Conclusions

We present ZINQ, a quantile-based approach to test taxon-level association of microbiota with dichotomous or quantitative clinical variables. Existing methods suffer from either inflated type I error or loss of power. The tailored methods for genomic or microbiome analysis usually impose strong parametric assumptions, which rarely hold due to the complex distributional attributes of microbiome data, leading to type I error inflation. Classical statistical methods such as linear regression and Wilcoxon tests control type I error but reduce testing power since they miss characteristics of microbiome data. We use the quantile regression framework, which is a robust non-parametric alternative, to handle the complicated distributional features of microbiome data. Also, by a comprehensive investigation of the association over different quantile levels of a taxon’s abundance, we can improve the testing power, and also detect complex mechanisms which might be of interest for biological researchers. Therefore, ZINQ provides a powerful and robust approach to microbiome differential abundance analysis, improving and complementing existing approaches.

## Appendix 1 – quantile rank-score based test with zero inflation

### Rank-score test for *β*(*τ*)=0 adjusting for zero inflation

Existing quantile regression inference tools will underestimate the uncertainty that the “non-zero subset” is observed by chance (i.e., underestimate the variance) and lead to biased tests. Therefore, we introduce a novel rank-score test of *β*(*τ*) to tackle zero inflation under the two-part quantile regression model.

Let $\widetilde C_{i} = C_{i} \cdot I (Y_{i} > 0)$ be the nominal clinical variable of interest, and let $\widetilde {\boldsymbol {Z}}_{i} = \boldsymbol {Z}_{i} \cdot I (Y_{i} > 0)$ be the nominal remaining covariates. Then $\widetilde {\boldsymbol {C}}_{n\times 1}=(\widetilde {C}_{1}, \cdots,\widetilde {C}_{n})^{\top }, \widetilde {\boldsymbol {Z}}_{n\times p}=(\widetilde {\boldsymbol {Z}}_{1}, \cdots, \widetilde {\boldsymbol {Z}}_{n})^{\top }$ are the design vector and matrix associated with $\widetilde {C}_{i}$’s and $\widetilde {\boldsymbol {Z}}_{i}$’s, respectively. We further define $ \widetilde {\boldsymbol {C}}^{*} = (\boldsymbol {I} - \widetilde {\boldsymbol {Z}} (\widetilde {\boldsymbol {Z}}^{\top } \widetilde {\boldsymbol {Z}})^{-1} \widetilde {\boldsymbol {Z}}^{\top }) \widetilde {\boldsymbol {C}}, $ where ***I*** is the *n*×*n* identity matrix. This transformation ensures that $\tilde {\boldsymbol {C}}^{*}$ exclusively contains the information about the clinical variable, since it is the vector of the least square residuals from regressing $\widetilde {\boldsymbol {C}}$ on $\widetilde {\boldsymbol {Z}}$. Then, we construct a rank score for *β*(*τ*)=0 by

5 $$ S^{Q}_{n, \tau} = n^{-\frac{1}{2}} \sum_{i = 1}^{n} \psi_{\tau} \{Y_{i} - \tilde {\boldsymbol{Z}}_{i}^{\top} \widehat{\boldsymbol{\alpha}}_{n} (\tau)\} I (Y_{i} > 0) \tilde {C}^{*}_{i},   $$

where *ψ*_*τ*_(*u*)=*τ*−*I*(*u*<0) is the piecewise first derivative of the quantile loss function $\rho _{\tau }(u), \widehat {\boldsymbol {\alpha }}_{n} (\tau)$ is the minimizer of (1) with *β*=0, and $\widetilde {C}_{i}^{*}$ is the *i*th element of $\widetilde {\boldsymbol {C}}^{*}$. $S^{Q}_{n, \tau } $ measures the independent contribution of the clinical variable to the *τ*th percentile of the non-zero normalized microbial abundance. As an analogy to the Rao’s score under likelihood-based models, it assesses the constraint *β*(*τ*)=0 based on the gradient of quantile loss function. When $\beta (\tau) = 0, S^{Q}_{n, \tau } $ is close to zero, while its substantial deviation from 0 indicates a significant effect of the clinical variable. Note that the zero-positive uncertainty is incorporated into the rank-score (5).

Finally, we define the rank-score test statistic at the *τ*th quantile as

6 $$ T^{Q}_{\tau} = \frac{S^{Q}_{n, \tau}}{\sqrt{ n^{-1} \tau (1 - \tau) \widetilde {\boldsymbol{C}}^{* \top} \widetilde {\boldsymbol{C}}^{*} }}.   $$

Under the null hypothesis (2), $T^{Q}_{\tau }$ asymptotically follows a standard normal distribution, and the *p*-value $p^{Q}_{\tau }$ can be obtained accordingly. The novel test has two major differences compared to the standard rank-score test. First, the rank-score (5) is computed based on the subset of data with non-zero *Y*_*i*_’s. Second, to correct the biases caused by zero inflation, we incorporate the zero-positive uncertainty in estimating the variance of the rank-score by introducing the zero-truncated nominal covariates. As $\mathbb {E}(\tilde { C}_{i}^{2}) = \mathbb {E}\{C_{i}^{2} P(Y_{i} >0 | \boldsymbol {X}_{i})\} $, the variance term in (6) implicitly incorporates a “propensity score” of each sample, compensating for the variability due to the random status of the taxon being sampled or not.

### Dependence structure of the novel rank-scores at multiple *τ*’s

We can compute a sequence of *p*-values $p^{Q}_{\tau _{k}}, k = 1, \cdots, K$ independently based on $T^{Q}_{\tau _{k}}, k=1, \cdots, K$ at the quantile levels 0<*τ*_1_<⋯<*τ*_*K*_<1. Next, under the null hypothesis (2), we can derive that $\boldsymbol {S}^{Q}_{n} = (S^{Q}_{n,\tau _{1}},\cdots,S^{Q}_{n,\tau _{K}})$ follows a multivariate normal distribution with mean ***0*** and covariance ***Σ***, where the (*k*,*k*)th diagonal element of ***Σ*** can be estimated by $n^{-1} \tau (1 - \tau) \widetilde {\boldsymbol {C}}^{* \top } \widetilde {\boldsymbol {C}}^{*}$, the (*k*,*l*)th off-diagonal element can be computed by $n^{-1} (\min \{\tau _{k}, \tau _{l}\} - \tau _{k} \, \tau _{l}) \tilde {\boldsymbol {C}}^{* \top } \tilde {\boldsymbol {C}}^{*}$. This test dependence structure will be used to combine the marginal *p*-values.

## Appendix 2 – resampling in MinP procedure

Let $q^{L}_{\text {min}}$ denote the (1−*T*_ZINQ-MinP_)th percentile of the distribution of *T*^*L*^, and $q^{Q}_{\text {min}}$ denote the (1−*T*_ZINQ-MinP_)th percentile of the distributions of $T^{Q}_{\tau _{k}}, k=1, \cdots, K$. The *p*-value based on *T*_ZINQ-MinP_ is

$$\begin{array}{@{}rcl@{}} && P \left\{ \,\, \exists \,\,\, T^{Q}_{\tau_{k}} \geq q^{Q}_{\text{min}}, k = 1, \cdots, K \,\, \text{or} \,\, T^{L} \geq q^{L}_{\text{min}} \,\, | \,\, H_{0} \,\, \right\} \\ &=& 1 - P \left\{\, T^{L} < q^{L}_{\text{min}} \, | \, H_{0} \,\right\}\\&& \, P \left\{\,\, \forall \,\,\, T^{Q}_{\tau_{k}} < q^{Q}_{\text{min}}, k = 1, \cdots, K \, | \, H_{0} \, \right\} \\ &=& 1 - (1 - T_{\text{ZINQ-MinP}})\\&& \, P \left\{\,\, \forall \,\,\, T^{Q}_{\tau_{k}} < q^{Q}_{\text{min}}, k = 1, \cdots, K \, | \, H_{0} \, \right\}, \end{array} $$

where the first equality is based on the conditional independence between *T*^*L*^ and $T^{Q}_{\tau }$. The joint probability $P \left \{ \forall \, T^{Q}_{\tau _{k}} < q^{Q}_{\text {min}}, k = 1, \cdots, K | H_{0} \right \}$ can be computed via resampling $S^{Q}_{n,\tau _{k}}$’s from the joint limiting distribution of $\boldsymbol {S}^{Q}_{n}$ under the null, and calculating the realizations of $T^{Q}_{\tau _{k}}$’s.

## Supplementary Information

**Additional file 1** PDF file includes supplemental tables (Tables S1–S18).

## Data Availability

Data used in this article is available from the CARDIA Study Data Coordinating Center at the University of Alabama at Birmingham. The process for obtaining data through CARDIA is outlined at https://www.cardia.dopm.uab.edu/publications-2/publications-documents. The R package ZINQ is available at https://github.com/wdl2459/ZINQ-v2 in formats appropriate for Macintosh or Windows systems. A vignette demonstrating use of the package is included (can be accessed at https://wdl2459.github.io/ZINQ-v2/ZINQ.Vignette.html).
