# Supplementary material for: Powerful and robust non-parametric association testing for microbiome data via a zero-inflated quantile approach (ZINQ)
Source: Microbiome. 2021 Sep 2;9:181. doi: 10.1186/s40168-021-01129-3 (PMC8414689; doi:10.1186/s40168-021-01129-3)
Supplement: Supplementary file 2 — Additional file 1 PDF file includes supplemental tables (Tables S1–S18). [file 40168_2021_1129_MOESM1_ESM.pdf]

Supplementary Material

Powerful and robust non-parametric association testing for microbiome data via a zero-inflated quantile approach (ZINQ)

Wodan Ling, Ni Zhao, Anna M. Plantinga, Lenore J. Launer, Anthony A. Fodor,
Katie A. Meyer and Michael C. Wu

The supplementary material includes additional tables for simulation studies, a table summarizing computational cost, and a table summarizing models, approaches and normalization methods used in the paper.

Table S1: Type I error control and power on 10000 simulated datasets generated from the edf of *Anaerovorax*'s normalized abundance with sample size 50, under different settings with significance cutoffs 0.05 and 0.01.

**sample size = 50**					
			Rarefaction		
	Type I error			Power	
	Null		Setting 1	Setting 2	Setting 3
α-level	0.05	0.01	0.05	0.05	0.05
ZIP	0.7123	0.6273	0.8359*	0.7360*	0.6789*
ZINB	0.1749	0.0600	0.4142*	0.2628*	0.1874*
linear regression	0.0329	0.0043	0.2632+	0.1088+	0.0402+
ZINQ-MinP	0.0398	0.0066	0.1965+	0.0839+	0.0428+
ZINQ-Cauchy	0.0392	0.0053	0.2186+	0.0888+	0.0448+
			TSS		
	Type I error			Power	
	Null		Setting 1	Setting 2	Setting 3
α-level	0.05	0.01	0.05	0.05	0.05
ZIB	0.0177	0.0032	0.0963+	0.0313+	0.0119+
Tobit	0.0449	0.0087	0.3154	0.1402	0.0557
ZIlogN	0.5128	0.5079	0.9467*	0.9328*	0.9174*
ZIG	0.0254	0.0023	0.1848+	0.0795+	0.0318+
linear regression	0.0332	0.0040	0.2559+	0.1070+	0.0403+
ZINQ-MinP	0.0328	0.0038	0.1896+	0.0781+	0.0367+
ZINQ-Cauchy	0.0363	0.0065	0.2412+	0.0954+	0.0408+
			CSS		
	Type I error			Power	
	Null		Setting 1	Setting 2	Setting 3
α-level	0.05	0.01	0.05	0.05	0.05
Tobit	0.0570	0.0153	0.3331	0.1543	0.0626
ZIlogN	0.0292	0.0047	0.2630+	0.0956+	0.0384+
ZIG	0.0268	0.0037	0.2356+	0.0864+	0.0341+
linear regression	0.0479	0.0119	0.3328	0.1445	0.0542
ZINQ-MinP	0.0327	0.0041	0.1641+	0.0731+	0.0380+
ZINQ-Cauchy	0.0363	0.0065	0.2096+	0.0865+	0.0405+

Setting 1: 100% from HBP edf for HBP samples;
Setting 2: 80% from HBP edf and 20% from non-HBP edf for HBP samples;
Setting 3: 60% from HBP edf and 40% from non-HBP edf for HBP samples.
*: power of a method that inflates type I error
+: power of a method that deflates type I error

Table S2: Type I error control and power on 10000 simulated datasets generated from the edf of *Anaerovorax*'s normalized abundance with sample size 100, under different settings with significance cutoffs 0.05 and 0.01.

**sample size = 100**					
			Rarefaction		
	Type I error		Power		
	Null		Setting 1	Setting 2	Setting 3
α-level	0.05	0.01	0.05	0.05	0.05
ZIP	0.7222	0.6428	0.9314*	0.8115*	0.7031*
ZINB	0.1862	0.0661	0.5969*	0.3538*	0.2067*
linear regression	0.0367	0.0044	0.4916^{+}	0.1927^{+}	0.0486^{+}
ZINQ-MinP	0.0465	0.0075	0.4552	0.1748	0.0590
ZINQ-Cauchy	0.0474	0.0076	0.5063	0.1880	0.0622
			TSS		
	Type I error		Power		
	Null		Setting 1	Setting 2	Setting 3
α-level	0.05	0.01	0.05	0.05	0.05
ZIB	0.0151	0.0020	0.2274^{+}	0.0576^{+}	0.0146^{+}
Tobit	0.0446	0.0070	0.5490	0.2252	0.0615
ZIlogN	0.5375	0.5233	0.9904*	0.9842*	0.9783*
ZIG	0.0443	0.0063	0.4273	0.1639	0.0551
linear regression	0.0360	0.0040	0.4788^{+}	0.1863^{+}	0.0498^{+}
ZINQ-MinP	0.0433	0.0083	0.4940	0.1981	0.0565
ZINQ-Cauchy	0.0485	0.0082	0.5578	0.2260	0.0613
			CSS		
	Type I error		Power		
	Null		Setting 1	Setting 2	Setting 3
α-level	0.05	0.01	0.05	0.05	0.05
Tobit	0.0549	0.0135	0.5602	0.2365	0.0713
ZIlogN	0.0441	0.0062	0.5491	0.2079	0.0569
ZIG	0.0427	0.0063	0.5394	0.1984	0.0557
linear regression	0.0508	0.0115	0.5929	0.2464	0.0674
ZINQ-MinP	0.0436	0.0083	0.4638	0.1815	0.0556
ZINQ-Cauchy	0.0485	0.0082	0.5068	0.2017	0.0593

Setting 1: 100% from HBP edf for HBP samples;
Setting 2: 80% from HBP edf and 20% from non-HBP edf for HBP samples;
Setting 3: 60% from HBP edf and 40% from non-HBP edf for HBP samples.
*: power of a method that inflates type I error
$^{+}$: power of a method that deflates type I error

Table S3: Type I error control and power on 10000 simulated datasets generated from the edf of *Anaerovorax*'s normalized abundance with sample size 200, under different settings with significance cutoffs 0.05 and 0.01.

**sample size = 200**					
			Rarefaction		
	Type I error		Power		
	Null		Setting 1	Setting 2	Setting 3
α-level	0.05	0.01	0.05	0.05	0.05
ZIP	0.7378	0.6644	0.9822*	0.8763*	0.7300*
ZINB	0.1942	0.0712	0.7785*	0.4774*	0.2307*
linear regression	0.0434	0.0057	0.7567	0.3365	0.0675
ZINQ-MinP	0.0473	0.0102	0.7889	0.3394	0.0733
ZINQ-Cauchy	0.0522	0.0101	0.8406	0.3862	0.0802
			TSS		
	Type I error		Power		
	Null		Setting 1	Setting 2	Setting 3
α-level	0.05	0.01	0.05	0.05	0.05
ZIB	0.0129	0.0017	0.5800$^+$	0.1384$^+$	0.0161$^+$
Tobit	0.0472	0.0070	0.8189	0.3838	0.0747
ZIlogN	0.5786	0.5613	0.9996*	0.9988*	0.9979*
ZIG	0.0543	0.0095	0.7300	0.3057	0.0764
linear regression	0.0439	0.0055	0.7620	0.3329	0.0677
ZINQ-MinP	0.0416	0.0075	0.8333	0.3646	0.0727
ZINQ-Cauchy	0.0499	0.0079	0.8742	0.4284	0.0856
			CSS		
	Type I error		Power		
	Null		Setting 1	Setting 2	Setting 3
α-level	0.05	0.01	0.05	0.05	0.05
Tobit	0.0543	0.0128	0.8343	0.4164	0.0902
ZIlogN	0.0502	0.0087	0.8520	0.4019	0.0788
ZIG	0.0478	0.0087	0.8572	0.3984	0.0745
linear regression	0.0525	0.0116	0.8657	0.4428	0.0887
ZINQ-MinP	0.0496	0.0081	0.7967	0.3470	0.0733
ZINQ-Cauchy	0.0498	0.0079	0.8416	0.3863	0.0802

Setting 1: 100% from HBP edf for HBP samples;
Setting 2: 80% from HBP edf and 20% from non-HBP edf for HBP samples;
Setting 3: 60% from HBP edf and 40% from non-HBP edf for HBP samples.
*: power of a method that inflates type I error
$^+$: power of a method that deflates type I error

Table S4: Type I error control and power on 10000 simulated datasets generated from the edf of *Saccharibacteria*'s normalized abundance with sample size 50, under different settings with significance cutoffs 0.05 and 0.01.

**sample size = 50**					
		Rarefaction			
	Type I error		Power		
	Null		Setting 1	Setting 2	Setting 3
α-level	0.05	0.01	0.05	0.05	0.05
ZIP	0.6745	0.5866	0.6456*	0.6324*	0.6285*
ZINB	0.2091	0.1124	0.1692*	0.1612*	0.1516*
linear regression	0.0339	0.0039	0.0546^{+}	0.0453^{+}	0.0409^{+}
ZINQ-MinP	0.0375	0.0062	0.0603^{+}	0.0473^{+}	0.0393^{+}
ZINQ-Cauchy	0.0364	0.0053	0.0602^{+}	0.0494^{+}	0.0430^{+}
		TSS			
	Type I error		Power		
	Null		Setting 1	Setting 2	Setting 3
α-level	0.05	0.01	0.05	0.05	0.05
ZIB	0.0040	0.0000	0.0079^{+}	0.0075^{+}	0.0071^{+}
Tobit	0.0427	0.0056	0.0579	0.0510	0.0487
ZIlogN	0.8939	0.8922	0.7540*	0.7606*	0.7639*
ZIG	0.0114	0.0010	0.0237^{+}	0.0175^{+}	0.0151^{+}
linear regression	0.0317	0.0027	0.0506^{+}	0.0434^{+}	0.0383^{+}
ZINQ-MinP	0.0278	0.0046	0.0567^{+}	0.0390^{+}	0.0298^{+}
ZINQ-Cauchy	0.0316	0.0063	0.0579^{+}	0.0404^{+}	0.0321^{+}
		CSS			
	Type I error		Power		
	Null		Setting 1	Setting 2	Setting 3
α-level	0.05	0.01	0.05	0.05	0.05
Tobit	0.0573	0.0142	0.0706	0.0637	0.0588
ZIlogN	0.0153	0.0026	0.0399^{+}	0.0248^{+}	0.0163^{+}
ZIG	0.0143	0.0025	0.0364^{+}	0.0226^{+}	0.0160^{+}
linear regression	0.0483	0.0102	0.0634	0.0515	0.0475
ZINQ-MinP	0.0274	0.0047	0.0669^{+}	0.0416^{+}	0.0300^{+}
ZINQ-Cauchy	0.0316	0.0063	0.0690^{+}	0.0443^{+}	0.0317^{+}

Setting 1: 100% from HBP edf for HBP samples;
Setting 2: 80% from HBP edf and 20% from non-HBP edf for HBP samples;
Setting 3: 60% from HBP edf and 40% from non-HBP edf for HBP samples.
*: power of a method that inflates type I error
$^{+}$: power of a method that deflates type I error

Table S5: Type I error control and power on 10000 simulated datasets generated from the edf of *Saccharibacteria*'s normalized abundance with sample size 100, under different settings with significance cutoffs 0.05 and 0.01.

**sample size = 100**					
			Rarefaction		
	Type I error		Power		
	Null		Setting 1	Setting 2	Setting 3
α-level	0.05	0.01	0.05	0.05	0.05
ZIP	0.6972	0.6130	0.6559*	0.6421*	0.6367*
ZINB	0.2476	0.1591	0.1971*	0.1824*	0.1736*
linear regression	0.0373	0.0041	0.0572+	0.0465+	0.0421+
ZINQ-MinP	0.0426	0.0085	0.0847	0.0541	0.0412
ZINQ-Cauchy	0.0472	0.0086	0.0895	0.0604	0.0449
			TSS		
	Type I error		Power		
	Null		Setting 1	Setting 2	Setting 3
α-level	0.05	0.01	0.05	0.05	0.05
ZIB	0.0111	0.0006	0.0194+	0.0147+	0.0115+
Tobit	0.0379	0.0058	0.0556+	0.0473+	0.0442+
ZIlogN	0.9699	0.9690	0.8595*	0.8603*	0.8594*
ZIG	0.0261	0.0027	0.0416+	0.0337+	0.0282+
linear regression	0.0323	0.0039	0.0525+	0.0423+	0.0386+
ZINQ-MinP	0.0385	0.0078	0.1101+	0.0637+	0.0396+
ZINQ-Cauchy	0.0439	0.0088	0.1317	0.0736	0.0445
			CSS		
	Type I error		Power		
	Null		Setting 1	Setting 2	Setting 3
α-level	0.05	0.01	0.05	0.05	0.05
Tobit	0.0562	0.0138	0.0742	0.0594	0.0549
ZIlogN	0.0252	0.0040	0.0956+	0.0491+	0.0291+
ZIG	0.0242	0.0033	0.0901+	0.0460+	0.0297+
linear regression	0.0509	0.0119	0.0730	0.0562	0.0513
ZINQ-MinP	0.0384	0.0078	0.1767+	0.0824+	0.0428+
ZINQ-Cauchy	0.0421	0.0085	0.1745	0.0860	0.0450

Setting 1: 100% from HBP edf for HBP samples;

Setting 2: 80% from HBP edf and 20% from non-HBP edf for HBP samples;

Setting 3: 60% from HBP edf and 40% from non-HBP edf for HBP samples.

*: power of a method that inflates type I error

+: power of a method that deflates type I error

Table S6: Type I error control and power on 10000 simulated datasets generated from the edf of *Saccharibacteria*'s normalized abundance with sample size 200, under different settings with significance cutoffs 0.05 and 0.01.

**sample size = 200**					
			Rarefaction		
	Type I error		Power		
	Null		Setting 1	Setting 2	Setting 3
α-level	0.05	0.01	0.05	0.05	0.05
ZIP	0.7361	0.6695	0.6909*	0.6757*	0.6682*
ZINB	0.3236	0.2256	0.2453*	0.2332*	0.2215*
linear regression	0.0353	0.0056	0.0622+	0.0502+	0.0417+
ZINQ-MinP	0.0463	0.0082	0.1575	0.0862	0.0535
ZINQ-Cauchy	0.0510	0.0086	0.1712	0.0945	0.0535
			TSS		
	Type I error		Power		
	Null		Setting 1	Setting 2	Setting 3
α-level	0.05	0.01	0.05	0.05	0.05
ZIB	0.0093	0.0018	0.0248+	0.0141+	0.0086+
Tobit	0.0358	0.0046	0.0510+	0.0466+	0.0425+
ZIlogN	0.9971	0.9967	0.9466*	0.9436*	0.9414*
ZIG	0.0429	0.0055	0.0670	0.0534	0.0452
linear regression	0.0315	0.0046	0.0486+	0.0440+	0.0402+
ZINQ-MinP	0.0428	0.0073	0.2218	0.0958	0.0466
ZINQ-Cauchy	0.0477	0.0082	0.2475	0.1158	0.0556
			CSS		
	Type I error		Power		
	Null		Setting 1	Setting 2	Setting 3
α-level	0.05	0.01	0.05	0.05	0.05
Tobit	0.0535	0.0123	0.0950	0.0687	0.0561
ZIlogN	0.0400	0.0060	0.2026	0.0920	0.0451
ZIG	0.0402	0.0057	0.2088	0.0956	0.0479
linear regression	0.0510	0.0113	0.1031	0.0703	0.0540
ZINQ-MinP	0.0382	0.0077	0.3529+	0.1369+	0.0488+
ZINQ-Cauchy	0.0459	0.0095	0.3533	0.1443	0.0560

Setting 1: 100% from HBP edf for HBP samples;
Setting 2: 80% from HBP edf and 20% from non-HBP edf for HBP samples;
Setting 3: 60% from HBP edf and 40% from non-HBP edf for HBP samples.
*: power of a method that inflates type I error
+: power of a method that deflates type I error

Table S7: Type I error control and power on 10000 simulated datasets generated from the edf of *Propionispira*'s normalized abundance with sample size 600, under different settings with significance cutoffs 0.05 and 0.01.

**Propionispira**					
			Rarefaction		
	Type I error			Power	
	Null		Setting 1	Setting 2	Setting 3
α-level	0.05	0.01	0.05	0.05	0.05
ZIP	0.7145	0.6359	0.9812*	0.8815*	0.7683*
ZINB	0.1831	0.0522	0.9055*	0.5545*	0.2454*
linear regression	0.0477	0.0048	0.8203	0.3546	0.0815
ZINQ-MinP	0.0457	0.0064	0.6676	0.2597	0.0660
ZINQ-Cauchy	0.0435	0.0074	0.7259	0.3070	0.0748
			TSS		
	Type I error			Power	
	Null		Setting 1	Setting 2	Setting 3
α-level	0.05	0.01	0.05	0.05	0.05
ZIB	0.0125	0.0022	0.6267+	0.2110+	0.0409+
Tobit	0.0458	0.0067	0.8859	0.4482	0.0888
ZIlogN	0.9003	0.8964	0.8399*	0.5318*	0.3659*
ZIG	0.0452	0.0081	0.8495	0.3724	0.0802
linear regression	0.0486	0.0058	0.8507	0.3750	0.0833
ZINQ-MinP	0.0465	0.0082	0.7051	0.2766	0.0680
ZINQ-Cauchy	0.0459	0.0070	0.8176	0.3879	0.0813
			CSS		
	Type I error			Power	
	Null		Setting 1	Setting 2	Setting 3
α-level	0.05	0.01	0.05	0.05	0.05
Tobit	0.0433	0.0063	0.8311	0.4043	0.0841
ZIlogN	0.0439	0.0083	0.7339	0.3162	0.0714
ZIG	0.0423	0.0078	0.7838	0.3390	0.0715
linear regression	0.0517	0.0109	0.8670	0.4412	0.0922
ZINQ-MinP	0.0467	0.0082	0.7195	0.2836	0.0679
ZINQ-Cauchy	0.0459	0.0070	0.8254	0.3904	0.0819

Setting 1: 100% from HBP edf for HBP samples;
Setting 2: 80% from HBP edf and 20% from non-HBP edf for HBP samples;
Setting 3: 60% from HBP edf and 40% from non-HBP edf for HBP samples.
*: power of a method that inflates type I error
+: power of a method that deflates type I error

Table S8: Type I error control and power on 10000 simulated datasets generated from the edf of *Corynebacterium*'s normalized abundance with sample size 600, under different settings with significance cutoffs 0.05 and 0.01.

**Corynebacterium**					
			Rarefaction		
	Type I error		Power		
	Null		Setting 1	Setting 2	Setting 3
α-level	0.05	0.01	0.05	0.05	0.05
ZIP	0.3911	0.2455	0.6017*	0.4351*	0.3364*
ZINB	0.2655	0.1137	0.4759*	0.3065*	0.2170*
linear regression	0.0333	0.0045	0.0658+	0.0513+	0.0409+
ZINQ-MinP	0.0395	0.0048	0.1892+	0.0797+	0.0470+
ZINQ-Cauchy	0.0434	0.0063	0.2639	0.1145	0.0486
			TSS		
	Type I error		Power		
	Null		Setting 1	Setting 2	Setting 3
α-level	0.05	0.01	0.05	0.05	0.05
ZIB	0.0051	0.0004	0.0057+	0.0057+	0.0059+
Tobit	0.0490	0.0080	0.0587	0.0523	0.0485
ZIlogN	0.2228	0.1476	0.4215*	0.2770*	0.1934*
ZIG	0.0563	0.0087	0.1057	0.0804	0.0612
linear regression	0.0483	0.0060	0.0969	0.0655	0.0494
ZINQ-MinP	0.0396	0.0063	0.6445+	0.2323+	0.0516+
ZINQ-Cauchy	0.0464	0.0081	0.3814	0.1102	0.0471
			CSS		
	Type I error		Power		
	Null		Setting 1	Setting 2	Setting 3
α-level	0.05	0.01	0.05	0.05	0.05
Tobit	0.0474	0.0074	0.0524	0.0466	0.0453
ZIlogN	0.0464	0.0078	0.2293	0.1037	0.0519
ZIG	0.0480	0.0097	0.1937	0.0972	0.0534
linear regression	0.0521	0.0106	0.0870	0.0635	0.0525
ZINQ-MinP	0.0468	0.0087	0.7047	0.2805	0.0664
ZINQ-Cauchy	0.0459	0.0081	0.4510	0.1288	0.0477

Setting 1: 100% from HBP edf for HBP samples;
Setting 2: 80% from HBP edf and 20% from non-HBP edf for HBP samples;
Setting 3: 60% from HBP edf and 40% from non-HBP edf for HBP samples.
*: power of a method that inflates type I error
+: power of a method that deflates type I error

Table S9: Average FPR and TPR by adjusted analysis on CLR transformed simulated OTU tables with sample size 531 generated from the two-part quantile model fitted on CARDIA data, over 1000 runs according to significance cutoffs 0.05 and 0.01.

**sample size = 531**				
	CLR		*CLR with zeroes*	
	FPR			
α-level	0.05	0.01	0.05	0.01
corncob	-	-	-	-
DESeq2	-	-	-	-
edgeR	-	-	-	-
LDM	0.0484	0.0099	0.0493	0.0101
limma	-	-	-	-
linear regression	0.0488	0.0099	0.0497	0.0100
metagenomeSeq	-	-	-	-
Monocle	-	-	-	-
QRank	0.0487	0.0089	0.0486	0.0096
ZINQ-MinP	0.0418	0.0082	0.0479	0.0092
ZINQ-Cauchy	0.0540	0.0107	0.0515	0.0101
	TPR			
α-level	0.05	0.01	0.05	0.01
corncob	-	-	-	-
DESeq2	-	-	-	-
edgeR	-	-	-	-
LDM	0.3975	0.2637	0.2864	0.1631
limma	-	-	-	-
linear regression	0.3986	0.2639	0.2874	0.1630
metagenomeSeq	-	-	-	-
Monocle	-	-	-	-
QRank	0.5853	0.4152	0.3189	0.1726
ZINQ-MinP	0.5410	0.3625	0.4422	0.2659
ZINQ-Cauchy	0.5844	0.4000	0.4778	0.2963

*: power of a method that inflates type I error

$^{+}$: power of a method that deflates type I error

Table S10: Average FPR and TPR by adjusted analysis on un-normalized/normalized simulated OTU tables with sample size 50 generated from the two-part quantile model fitted on CARDIA data, over 1000 runs according to significance cutoffs 0.05 and 0.01.

**sample size = 50**								
	Count		*Rarefaction*		*TSS*		*CSS*	
	FPR							
α-level	0.05	0.01	0.05	0.01	0.05	0.01	0.05	0.01
corncob	0.1652	0.0849	0.1606	0.0792	-	-	-	-
DESeq2	0.0553	0.0185	0.0488	0.0158	-	-	-	-
edgeR	0.0845	0.0297	0.0795	0.0262	-	-	-	-
LDM	0.0470	0.0085	0.0464	0.0086	0.0470	0.0085	0.0487	0.0097
limma	0.0605	0.0141	0.0706	0.0183	-	-	-	-
linear regression	0.0400	0.0068	0.0394	0.0064	0.0395	0.0065	0.0486	0.0095
metagenomeSeq	-	-	-	-	-	-	0.1613	0.0761
Monocle	0.7021	0.6414	0.6460	0.5797	0.0569	0.0114	0.0624	0.0145
QRank	0.0453	0.0073	0.0460	0.0077	0.0458	0.0073	0.0437	0.0071
ZINQ-MinP	0.0516	0.0095	0.0525	0.0096	0.0793	0.0233	0.0568	0.0110
ZINQ-Cauchy	0.0516	0.0089	0.0489	0.0082	0.0542	0.0123	0.0532	0.0094
	TPR							
α-level	0.05	0.01	0.05	0.01	0.05	0.01	0.05	0.01
corncob	0.2048*	0.1080*	0.1984*	0.1017*	-	-	-	-
DESeq2	0.0877	0.0356*	0.0736	0.0290*	-	-	-	-
edgeR	0.1266*	0.0516*	0.1165*	0.0446*	-	-	-	-
LDM	0.0748	0.0176	0.0722	0.0169	0.0748	0.0176	0.0952	0.0262
limma	0.1020*	0.0329*	0.1084*	0.0352*	-	-	-	-
linear regression	0.0668	0.0136+	0.0623+	0.0124+	0.0645+	0.0133+	0.0933	0.0257
metagenomeSeq	-	-	-	-	-	-	0.2105*	0.1058*
Monocle	0.7380*	0.6803*	0.6811*	0.6159*	0.0879	0.0216	0.1136*	0.0359*
QRank	0.0690	0.0143+	0.0639	0.0130+	0.0693	0.0143+	0.0649	0.0133+
ZINQ-MinP	0.0769	0.0159	0.0710	0.0141	0.0987*	0.0288*	0.0786	0.0170
ZINQ-Cauchy	0.0848	0.0181	0.0740	0.0150	0.0810	0.0204*	0.0842	0.0182

*: power of a method that inflates type I error

+: power of a method that deflates type I error

Table S11: Average FPR and TPR by adjusted analysis on un-normalized/normalized simulated OTU tables with sample size 100 generated from the two-part quantile model fitted on CARDIA data, over 1000 runs according to significance cutoffs 0.05 and 0.01.

**sample size = 100**								
	Count		*Rarefaction*		*TSS*		*CSS*	
				FPR				
α-level	0.05	0.01	0.05	0.01	0.05	0.01	0.05	0.01
corncob	0.1214	0.0569	0.1159	0.0526	-	-	-	-
DESeq2	0.0645	0.0225	0.0553	0.0186	-	-	-	-
edgeR	0.0895	0.0316	0.0818	0.0267	-	-	-	-
LDM	0.0486	0.0094	0.0492	0.0095	0.0486	0.0094	0.0496	0.0094
limma	0.0582	0.0136	0.0712	0.0190	-	-	-	-
linear regression	0.0423	0.0069	0.0424	0.0072	0.0420	0.0069	0.0499	0.0097
metagenomeSeq	-	-	-	-	-	-	0.1634	0.0826
Monocle	0.7172	0.6586	0.6544	0.5897	0.0514	0.0094	0.0564	0.0122
QRank	0.0479	0.0090	0.0490	0.0094	0.0482	0.0092	0.0470	0.0089
ZINQ-MinP	0.0527	0.0104	0.0550	0.0106	0.0536	0.0102	0.0505	0.0097
ZINQ-Cauchy	0.0543	0.0101	0.0530	0.0097	0.0545	0.0102	0.0539	0.0104
				TPR				
α-level	0.05	0.01	0.05	0.01	0.05	0.01	0.05	0.01
corncob	0.1948*	0.0979*	0.1812*	0.0866*	-	-	-	-
DESeq2	0.1262*	0.0597*	0.1042	0.0455*	-	-	-	-
edgeR	0.1711*	0.0793*	0.1527*	0.0663*	-	-	-	-
LDM	0.1086	0.0305	0.1027	0.0282	0.1086	0.0305	0.1430	0.0505
limma	0.1454	0.0543*	0.1458*	0.0545*	-	-	-	-
linear regression	0.1000	0.0247$^+$	0.0909	0.0213$^+$	0.0957	0.0230$^+$	0.1404	0.0498
metagenomeSeq	-	-	-	-	-	-	0.2614*	0.1481*
Monocle	0.7727*	0.7196*	0.7096*	0.6478*	0.1115	0.0297	0.1524	0.0571*
QRank	0.1044	0.0289	0.0913	0.0234	0.1015	0.0287	0.0996	0.0273
ZINQ-MinP	0.1171	0.0328	0.1020	0.0263	0.1141	0.0314	0.1125	0.0315
ZINQ-Cauchy	0.1375	0.0415	0.1184	0.0324	0.1339	0.0398	0.1328	0.0392

*: power of a method that inflates type I error

$^+$: power of a method that deflates type I error

Table S12: Average FPR and TPR by adjusted analysis on un-normalized/normalized simulated OTU tables with sample size 200 generated from the two-part quantile model fitted on CARDIA data, over 1000 runs according to significance cutoffs 0.05 and 0.01.

**sample size = 200**								
	Count		*Rarefaction*		*TSS*		*CSS*	
				FPR				
α-level	0.05	0.01	0.05	0.01	0.05	0.01	0.05	0.01
corncob	0.1074	0.0506	0.0976	0.0429	-	-	-	-
DESeq2	0.0748	0.0282	0.0633	0.0227	-	-	-	-
edgeR	0.0959	0.0365	0.0865	0.0298	-	-	-	-
LDM	0.0503	0.0093	0.0499	0.0095	0.0503	0.0093	0.0495	0.0097
limma	0.0574	0.0128	0.0722	0.0198	-	-	-	-
linear regression	0.0447	0.0073	0.0446	0.0074	0.0446	0.0072	0.0495	0.0098
metagenomeSeq	-	-	-	-	-	-	0.1601	0.0803
Monocle	0.7217	0.6643	0.6540	0.5901	0.0499	0.0085	0.0529	0.0108
QRank	0.0502	0.0097	0.0490	0.0096	0.0499	0.0096	0.0498	0.0093
ZINQ-MinP	0.0504	0.0100	0.0510	0.0097	0.0495	0.0100	0.0495	0.0097
ZINQ-Cauchy	0.0546	0.0111	0.0541	0.0102	0.0551	0.0110	0.0539	0.0112
				TPR				
α-level	0.05	0.01	0.05	0.01	0.05	0.01	0.05	0.01
corncob	0.2564*	0.1386*	0.2257*	0.1171*	-	-	-	-
DESeq2	0.1927*	0.1093*	0.1611*	0.0835*	-	-	-	-
edgeR	0.2472*	0.1368*	0.2194*	0.1122*	-	-	-	-
LDM	0.1703	0.0620	0.1592	0.0564	0.1703	0.0620	0.2292	0.1070
limma	0.2240	0.1067*	0.2137*	0.0988*	-	-	-	-
linear regression	0.1655	0.0563^{+}	0.1486	0.0465^{+}	0.1583	0.0517^{+}	0.2273	0.1071
metagenomeSeq	-	-	-	-	-	-	0.3383*	0.2153*
Monocle	0.8110*	0.7646*	0.7485*	0.6905*	0.1670	0.0571	0.2339	0.1123
QRank	0.1780	0.0699	0.1445	0.0514	0.1701	0.0644	0.1683	0.0638
ZINQ-MinP	0.2038	0.0806	0.1637	0.0582	0.1948	0.0770	0.1941	0.0764
ZINQ-Cauchy	0.2363	0.1013	0.1943	0.0745	0.2275	0.0960	0.2244	0.0954

*: power of a method that inflates type I error

$^{+}$: power of a method that deflates type I error

Table S13: Average FPR and TPR by unadjusted analysis on CLR transformed simulated OTU tables with sample size 531 generated from the Dirichlet-Multinomial models fitted on CARDIA data, over 1000 runs according to significance cutoffs 0.05 and 0.01.

**sample size = 531**				
	CLR		*CLR with zeroes*	
	FPR			
α-level	0.05	0.01	0.05	0.01
corncob	-	-	-	-
DESeq2	-	-	-	-
edgeR	-	-	-	-
LDM	0.0493	0.0099	0.0493	0.0097
limma	-	-	-	-
linear regression	0.0497	0.0099	0.0497	0.0097
metagenomeSeq	-	-	-	-
Monocle	-	-	-	-
QRank	0.0496	0.0100	0.0487	0.0097
ZINQ-MinP	0.0412	0.0082	0.0470	0.0092
ZINQ-Cauchy	0.0544	0.0109	0.0510	0.0101
	TPR			
α-level	0.05	0.01	0.05	0.01
corncob	-	-	-	-
DESeq2	-	-	-	-
edgeR	-	-	-	-
LDM	0.2889	0.1660	0.1949	0.0934
limma	-	-	-	-
linear regression	0.2903	0.1661	0.1962	0.0936
metagenomeSeq	-	-	-	-
Monocle	-	-	-	-
QRank	0.5059	0.3366	0.1997	0.0895
ZINQ-MinP	0.4503	0.2734	0.2432	0.1200
ZINQ-Cauchy	0.4828	0.3001	0.2856	0.1471

*: power of a method that inflates type I error

$^{+}$: power of a method that deflates type I error

Table S14: Average FPR and TPR by unadjusted analysis on un-normalized/normalized simulated OTU tables with sample size 50 generated from the Dirichlet-Multinomial models fitted on CARDIA data, over 1000 runs according to significance cutoffs 0.05 and 0.01.

**sample size = 50**								
	Count		*Rarefaction*		*TSS*		*CSS*	
				FPR				
α-level	0.05	0.01	0.05	0.01	0.05	0.01	0.05	0.01
corncob	0.0838	0.0312	0.0840	0.0312	-	-	-	-
DESeq2	0.1303	0.0585	0.1302	0.0583	-	-	-	-
edgeR	0.0727	0.0212	0.0726	0.0215	-	-	-	-
LDM	0.0490	0.0094	0.0490	0.0094	0.0490	0.0094	0.0498	0.0096
limma	0.0540	0.0123	0.0537	0.0124	-	-	-	-
linear regression	0.0360	0.0056	0.0360	0.0056	0.0361	0.0056	0.0493	0.0098
metagenomeSeq	-	-	-	-	-	-	0.1655	0.0778
Monocle	0.9464	0.9295	0.9462	0.9289	0.0425	0.0071	0.0558	0.0118
QRank	0.0444	0.0070	0.0449	0.0071	0.0437	0.0071	0.0452	0.0073
ZINQ-MinP	0.0411	0.0067	0.0414	0.0065	0.0419	0.0082	0.0420	0.0083
ZINQ-Cauchy	0.0443	0.0068	0.0443	0.0067	0.0436	0.0069	0.0437	0.0069
				TPR				
α-level	0.05	0.01	0.05	0.01	0.05	0.01	0.05	0.01
corncob	0.1020*	0.0383*	0.1019*	0.0385*	-	-	-	-
DESeq2	0.1487*	0.0701*	0.1486*	0.0698*	-	-	-	-
edgeR	0.0889*	0.0287*	0.0896*	0.0288*	-	-	-	-
LDM	0.0652	0.0144	0.0652	0.0144	0.0652	0.0144	0.0779	0.0203
limma	0.0808	0.0222*	0.0808	0.0224*	-	-	-	-
linear regression	0.0490$^+$	0.0086$^+$	0.0490$^+$	0.0085$^+$	0.0489$^+$	0.0085$^+$	0.0763	0.0197
metagenomeSeq	-	-	-	-	-	-	0.1881*	0.0912*
Monocle	0.9496*	0.9338*	0.9492*	0.9332*	0.0568	0.0104$^+$	0.0848	0.0232
QRank	0.0588	0.0109$^+$	0.0586	0.0110$^+$	0.0587	0.0112$^+$	0.0578	0.0109$^+$
ZINQ-MinP	0.0533	0.0101$^+$	0.0529	0.0099$^+$	0.0550	0.0116	0.0550	0.0114
ZINQ-Cauchy	0.0622	0.0113$^+$	0.0619	0.0112$^+$	0.0620	0.0115$^+$	0.0623	0.0115$^+$

*: power of a method that inflates type I error

$^+$: power of a method that deflates type I error

Table S15: Average FPR and TPR by unadjusted analysis on un-normalized/normalized simulated OTU tables with sample size 100 generated from the Dirichlet-Multinomial models fitted on CARDIA data, over 1000 runs according to significance cutoffs 0.05 and 0.01.

**sample size = 100**								
	Count		*Rarefaction*		*TSS*		*CSS*	
				FPR				
α-level	0.05	0.01	0.05	0.01	0.05	0.01	0.05	0.01
corncob	0.0666	0.0192	0.0668	0.0192	-	-	-	-
DESeq2	0.1142	0.0454	0.1137	0.0452	-	-	-	-
edgeR	0.0653	0.0166	0.0642	0.0167	-	-	-	-
LDM	0.0515	0.0100	0.0515	0.0100	0.0515	0.0100	0.0498	0.0094
limma	0.0510	0.0107	0.0506	0.0106	-	-	-	-
linear regression	0.0418	0.0067	0.0418	0.0067	0.0418	0.0067	0.0493	0.0096
metagenomeSeq	-	-	-	-	-	-	0.1522	0.0686
Monocle	0.9480	0.9311	0.9472	0.9303	0.0450	0.0076	0.0520	0.0108
QRank	0.0486	0.0100	0.0490	0.0096	0.0495	0.0094	0.0488	0.0092
ZINQ-MinP	0.0443	0.0079	0.0445	0.0077	0.0446	0.0078	0.0448	0.0076
ZINQ-Cauchy	0.0487	0.0088	0.0489	0.0087	0.0490	0.0088	0.0492	0.0088
				TPR				
α-level	0.05	0.01	0.05	0.01	0.05	0.01	0.05	0.01
corncob	0.1102*	0.0366*	0.1102*	0.0365*	-	-	-	-
DESeq2	0.1454*	0.0624*	0.1451*	0.0623*	-	-	-	-
edgeR	0.0877*	0.0260*	0.0876*	0.0260*	-	-	-	-
LDM	0.0773	0.0192	0.0774	0.0192	0.0773	0.0192	0.1031	0.0321
limma	0.1046	0.0331	0.1052	0.0334	-	-	-	-
linear regression	0.0651	0.0136+	0.0650	0.0136+	0.0651	0.0136+	0.1035	0.0320
metagenomeSeq	-	-	-	-	-	-	0.1951*	0.0954*
Monocle	0.9507*	0.9356*	0.9505*	0.9348*	0.0700	0.0149+	0.1076	0.0343
QRank	0.0812	0.0212	0.0814	0.0208	0.0812	0.0211	0.0788	0.0202
ZINQ-MinP	0.0760	0.0179+	0.0758	0.0176+	0.0759	0.0180+	0.0747	0.0170+
ZINQ-Cauchy	0.0921	0.0235	0.0922	0.0234	0.0922	0.0235	0.0903	0.0226

*: power of a method that inflates type I error

+: power of a method that deflates type I error

Table S16: Average FPR and TPR by unadjusted analysis on un-normalized/normalized simulated OTU tables with sample size 200 generated from the Dirichlet-Multinomial models fitted on CARDIA data, over 1000 runs according to significance cutoffs 0.05 and 0.01.

**sample size = 200**								
	Count		*Rarefaction*		*TSS*		*CSS*	
				FPR				
α-level	0.05	0.01	0.05	0.01	0.05	0.01	0.05	0.01
corncob	0.0580	0.0144	0.0578	0.0145	-	-	-	-
DESeq2	0.1030	0.0367	0.1030	0.0367	-	-	-	-
edgeR	0.0605	0.0142	0.0608	0.0139	-	-	-	-
LDM	0.0501	0.0101	0.0500	0.0101	0.0501	0.0101	0.0506	0.0101
limma	0.0507	0.0107	0.0503	0.0107	-	-	-	-
linear regression	0.0447	0.0077	0.0448	0.0076	0.0448	0.0077	0.0502	0.0104
metagenomeSeq	-	-	-	-	-	-	0.1437	0.0622
Monocle	0.9467	0.9300	0.9459	0.9288	0.0468	0.0081	0.0517	0.0110
QRank	0.0502	0.0098	0.0496	0.0095	0.0501	0.0101	0.0493	0.0098
ZINQ-MinP	0.0462	0.0090	0.0462	0.0090	0.0461	0.0090	0.0470	0.0091
ZINQ-Cauchy	0.0516	0.0097	0.0515	0.0096	0.0519	0.0097	0.0515	0.0096
				TPR				
α-level	0.05	0.01	0.05	0.01	0.05	0.01	0.05	0.01
corncob	0.1560	0.0601*	0.1558	0.0600*	-	-	-	-
DESeq2	0.1572*	0.0664*	0.1570*	0.0665*	-	-	-	-
edgeR	0.1023*	0.0321*	0.1021*	0.0319*	-	-	-	-
LDM	0.0971	0.0295	0.0972	0.0295	0.0971	0.0295	0.1515	0.0594
limma	0.1562	0.0616	0.1563	0.0620	-	-	-	-
linear regression	0.0896	0.0243+	0.0895	0.0243+	0.0895	0.0243+	0.1544	0.0607
metagenomeSeq	-	-	-	-	-	-	0.2263*	0.1175*
Monocle	0.9531*	0.9387*	0.9526*	0.9377*	0.0921	0.0253	0.1573	0.0626
QRank	0.1176	0.0388	0.1171	0.0380	0.1178	0.0389	0.1135	0.0363
ZINQ-MinP	0.1179	0.0389	0.1176	0.0387	0.1176	0.0388	0.1146	0.0371
ZINQ-Cauchy	0.1432	0.0512	0.1428	0.0510	0.1431	0.0514	0.1393	0.0481

*: power of a method that inflates type I error

+: power of a method that deflates type I error

Table S17: Computation time and memory cost to analyze normalized CARDIA data 10 times.

	Rarefaction		CSS	
	Time(min)	Memory(GB)	Time(min)	Memory(GB)
corncob	84.5	2.0	–	–
DESeq2	5.0	0.8	–	–
edgeR	2.5	1.2	–	–
LDM	7.5	2.1	16.5	2.1
limma	1.0	1.7	–	–
linear regression	1.0	1.3	4.0	1.3
metagenomeSeq	–	–	4.0	1.3
Monocle	2.0	1.3	5.5	2.1
QRank	4.0	1.3	4.0	1.3
ZINQ-MinP	5.5	2.1	5.5	2.1
ZINQ-Cauchy	4.5	1.3	4.5	1.3

Table S18: Distributions/models and normalization approaches for each comparison method.

	Normalization Methods					Analysis Approaches	
Model	None[a]	Rarefy	TSS	CSS	CLR	Single Taxon	OTU Table
Zero-inflated Poisson	✓	✓				ZIP regression	-
Zero-inflated neg. binomial	✓	✓				ZINB regression	-
Negative binomial	✓	✓				-	DESeq2<br>edgeR<br>Monocle[b]
Zero-inflated beta			✓			ZIB regression	-
Beta-binomial	✓	✓				-	Corncob
Tobit			✓	✓		Tobit regression	Monocle[b]
Zero-inflated lognormal			(✓)	✓		ZIlogN regression<br>(TSS or CSS)	metagenomeSeq<br>(CSS; no covariates)
Lognormal	✓	✓				-	Limma
Zero-inflated normal				✓	-	metagenomeSeq	 (covariates ok)
Zero-inflated gamma			✓	✓		ZIG regression	-
Normal	✓	✓	✓	✓	✓	Linear regression	Linear regression
Linear decomposition	✓	✓	✓	✓	✓	-	LDM
Quantile model	✓	✓	✓	✓	✓	ZINQ-MinP<br>ZINQ-Cauchy	QRank<br>ZINQ-MinP<br>ZINQ-Cauchy

[a]: Original data (normalization = "none") was considered only for OTU table analysis

[b]: Monocle model depends on normalization method
